# Signatures of functional bacteriome structure in a tropical direct-developing amphibian species

**DOI:** 10.1186/s42523-022-00188-7

**Published:** 2022-06-07

**Authors:** Renato A. Martins, Sasha E. Greenspan, Daniel Medina, Shannon Buttimer, Vanessa M. Marshall, Wesley J. Neely, Samantha Siomko, Mariana L. Lyra, Célio F. B. Haddad, Vinícius São-Pedro, C. Guilherme Becker

**Affiliations:** 1 Programa de Pós-Graduação em Conservação da Fauna, Universidade Federal de São Carlos, São Carlos, SP 13565-905 Brazil; 2 Department of Biological Sciences, The University of Alabama, Tuscaloosa, AL 35487 USA; 3 Sistema Nacional de Investigación, SENACYT, Building 205, City of Knowledge, Clayton, Panama Republic of Panama; 4 Department of Biology, The Pennsylvania State University, University Park, PA 16803 USA; 5 Department of Biodiversity and Aquaculture Center (CAUNESP), Universidade Estadual Paulista, Rio Claro, SP 13506-900 Brazil; 6 Centro de Ciências da Natureza, Universidade Federal de São Carlos, Campus Lagoa do Sino, Buri, SP 18290-000 Brazil

**Keywords:** Bacterial co-occurrence network analysis, *Batrachochytrium dendrobatidis*, Brazil’s Atlantic Forest, Ecological core, *Haddadus binotatus*, *Ischnocnema henselii*, Microbiome

## Abstract

**Background:**

Host microbiomes may differ under the same environmental conditions and these differences may influence susceptibility to infection. Amphibians are ideal for comparing microbiomes in the context of disease defense because hundreds of species face infection with the skin-invading microbe *Batrachochytrium dendrobatidis* (Bd), and species richness of host communities, including their skin bacteria (bacteriome), may be exceptionally high. We conducted a landscape-scale Bd survey of six co-occurring amphibian species in Brazil’s Atlantic Forest. To test the bacteriome as a driver of differential Bd prevalence, we compared bacteriome composition and co-occurrence network structure among the six focal host species.

**Results:**

Intensive sampling yielded divergent Bd prevalence in two ecologically similar terrestrial-breeding species, a group with historically low Bd resistance. Specifically, we detected the highest Bd prevalence in *Ischnocnema henselii* but no Bd detections in *Haddadus binotatus*. *Haddadus binotatus* carried the highest bacteriome alpha and common core diversity, and a modular network partitioned by negative co-occurrences, characteristics associated with community stability and competitive interactions that could inhibit Bd colonization.

**Conclusions:**

Our findings suggest that community structure of the bacteriome might drive Bd resistance in *H. binotatus*, which could guide microbiome manipulation as a conservation strategy to protect diverse radiations of direct-developing species from Bd-induced population collapses.

**Supplementary Information:**

The online version contains supplementary material available at 10.1186/s42523-022-00188-7.

## Background

One of the most fundamental questions in disease ecology is: in a given host-microbe encounter, why do some individuals get sick while others do not? When germ theory gained acceptance in the late nineteenth century, research centered on highly transmissible, toxin-producing bacteria that consistently caused disease (e.g., diphtheria and cholera), leading to the microbe-centric view that virulence is a fixed microbial trait [1]. The role of the host gained traction in the twentieth century when large-scale disease emergences in immune-impaired human patients were attributed to microbes considered to be commensal. For example, widespread use of antibiotics increased oral candidiasis by the common fungal gut microbe *Candida albicans* [2] and patients immunocompromised from HIV were more susceptible to pneumococcal pneumonia from the common respiratory tract bacteria *Streptococcus pneumoniae* [3]. However, even this more host-centric view failed to account for a role of the host in disease outcomes in apparently healthy patients [4].

To better integrate microbe and host contributions to disease, the ‘damage-response framework’ of microbial pathogenicity rejects defining microbes as pathogens, commensals, or opportunists, instead defining only microbes and hosts which interact [5]. Microbial pathogenicity is then characterized by placing these context-dependent interactions on a continuum of host damage arising from the microbe, the host response, or both, and ranging from beneficial or neutral interactions to a level of cell, tissue, or organ damage that disrupts homeostasis, causing disease [6]. Eleven interrelated factors governing the outcome of these host-microbe interactions have been proposed, with the first letter of each factor assembling into the acronym MISTEACHING to underscore the rapidly evolving and complex nature of the field: microbiome, inoculum, sex, temperature, environment, age, chance, history, immunity, nutrition, and genetics [7]. While this list was originally proposed for human hosts, it is also applicable to other vertebrates.

As new approaches for studying microbes have exposed the host microbiome as a central player in host physiology, immunity, and health [8], the damage-response framework has been revised to consider the host component as a new entity encompassing the host and its microbiota [7]. Assembly of the host microbiome is a combination of deterministic and stochastic processes and can be viewed through a metacommunity lens: at the scale of the host individual, communities are influenced by host behavior and physiology, including immunity and chemical conditions, with individual communities linked through dispersal among conspecifics, heterospecifics, and free-living microbial communities, all against the backdrop of abiotic environmental conditions [9, 10]. In turn, host-associated microbes may mediate host interactions with invading microbes, and subsequent host damage, by stimulating the host immune system, competing with microbial intruders, or producing bioactive compounds [11].

Amphibians are an ideal system to study the spectrum of host responses to a given microbial species. Not only are they the most threatened vertebrate taxon [12], but they are also hosts to the skin-infecting chytrid fungus *Batrachochytrium dendrobatidis* (Bd), a microbe with a deadly combination of the greatest known vertebrate host breadth and, in hundreds of species, a high capacity for host damage [13]. The amphibian skin tissue that Bd infects is highly biologically active. In addition to the skin’s role in breathing and homeostasis, it also produces mucus secretions and chemical compounds that create distinct microhabitats for diverse host-associated bacterial communities (bacteriome) [14] known to influence host-Bd interactions [15–19]. Extreme variation in Bd pathogenicity has been reported across hundreds of tested host species, with many examples of host bacteriome composition correlating with Bd pathogenicity [20, 21]. For example, inoculations with Bd-inhibitory bacteria or bacterial metabolites increased survival in mountain yellow-legged frogs *Rana muscosa* [22], red-backed salamanders *Plethodon cinereus* [23] and boreal toads *Anaxyrus boreas* [24], and higher skin bacterial diversity was associated with lower Bd loads in the coqui frog *Eleutherodactylus coqui* [25].

Most other documented host contributors to Bd pathogenicity match the attributes of MISTEACHING: temperature [26–28], environmental contamination [29, 30], life stage [31, 32], behavior [33–35], acquired immunity [36], and innate immunity including MHC class II expression and genetic diversity [37, 38] and antimicrobial skin peptides [18, 39], with many of these factors interrelated with the bacteriome. An additional factor especially relevant to amphibians is host community structure. Amphibian communities can reach high levels of species richness relative to other terrestrial vertebrates, especially in the tropics, and often exhibit extreme within-community variation in life history strategies and other traits that may result in widely divergent host-microbe interactions, both from the perspective of the host bacteriome and invading microbes [40–42].

**Fig. 1:**
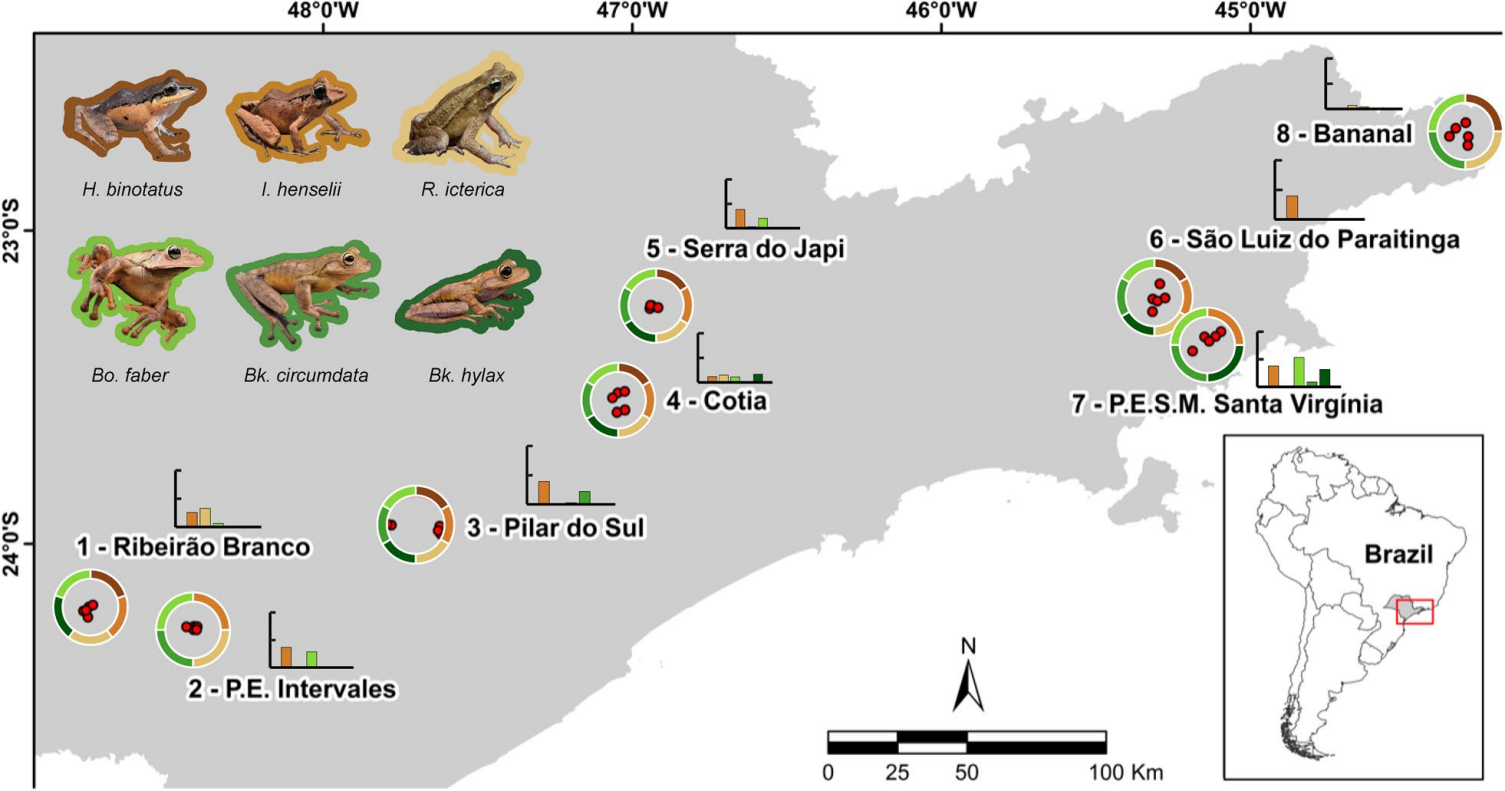
Occurrence of six focal amphibian species (shown at top left) in eight focal landscapes (circles of 15-km diameter) and 40 sample sites (five red points per landscape) in the Atlantic Forest in the State of São Paulo, southeastern Brazil. Colored bars within circles indicate species sampled within each landscape. Barplots show relative *Batrachochytrium dendrobatidis* prevalence among species observed at each landscape. Landscapes are (1) Ribeirão Branco, (2) Intervales State Park, (3) Pilar do Sul, (4) Cotia, (5) Serra do Japi, (6) São Luíz do Paraitinga, (7) Serra do Mar State Park—Núcleo Santa Virgínia, and (8) Bananal

Brazil’s Atlantic Forest supports a megadiverse fauna of amphibians [43] and Bd is generally widespread in the region [44]. As an aquatic pathogen, Bd is easily transmitted among aquatic-breeding frogs during seasonal breeding aggregations in water bodies [45–47]. In contrast, terrestrial-breeding species exhibiting direct development (i.e., without a free-living, aquatic larval stage; individuals hatch as froglets from terrestrial eggs) are rarely exposed to water bodies, in many cases translating to low immunity against Bd, but may encounter the pathogen sporadically through spillover from aquatic-breeding species or in response to climate anomalies [40, 48]. These terrestrial host-Bd interactions typically score high on the host damage continuum including high infection loads and mortality rates [48, 49]. We investigated Bd infection patterns in six Atlantic Forest anuran species. Four species, *Boana faber* (Hylidae), *Bokermannohyla circumdata* (Hylidae), *Bokermannohyla hylax* (Hylidae), and *Rhinella ornata* (Bufonidae) are aquatic-breeding, and two species, *Haddadus binotatus* (Craugastoridae) and *Ischnocnema henselii* (Brachycephalidae), are terrestrial-breeding. While we expected similar Bd infection patterns in the two ecologically similar terrestrial-breeding species, intensive sampling of 40 natural forest sites across eight landscapes yielded no detections of Bd-positive individuals of *H. binotatus* but substantial levels of infection in the other species, including relatively high Bd prevalence in *Ischnocnema henselii* (Fig. 1). Previous community-wide Bd surveys suggest that in habitats where sympatric species are infected, consistent non-detection of Bd in a single host species after exhaustive sampling is rare [40]. In addition, previous studies on Atlantic Forest amphibian assemblages indicate that the two terrestrial-breeding species experience similar probability of Bd exposure through spillover from aquatic-breeding species [48]. Together, these findings suggest that host factors such as the bacteriome may be the primary drivers of differential host damage between *H. binotatus* and *I. henselii*.

To test the hypothesis that the observed variation in Bd prevalence was associated with host bacterial communities, we compared bacteriome composition and structure among the six focal amphibian species, including alpha and beta diversity, a metric of dysbiosis known as dispersion, and co-occurrence network topology. If bacteriomes drive variation in host damage in this system, we would expect distinct bacteriome metrics in *H. binotatus* compared to the other species, especially with regard to characteristics previously associated with bacteriome stability and pathogen inhibition such as high microbial diversity [50], low dispersion [51], and strongly nonrandom network topology [52, 53]. Combined, our results shed new light on the potential role of bacteriome structure in microbial defense in historically understudied tropical amphibians.

## Results

### Bd prevalence and infection loads

**Fig. 2:**
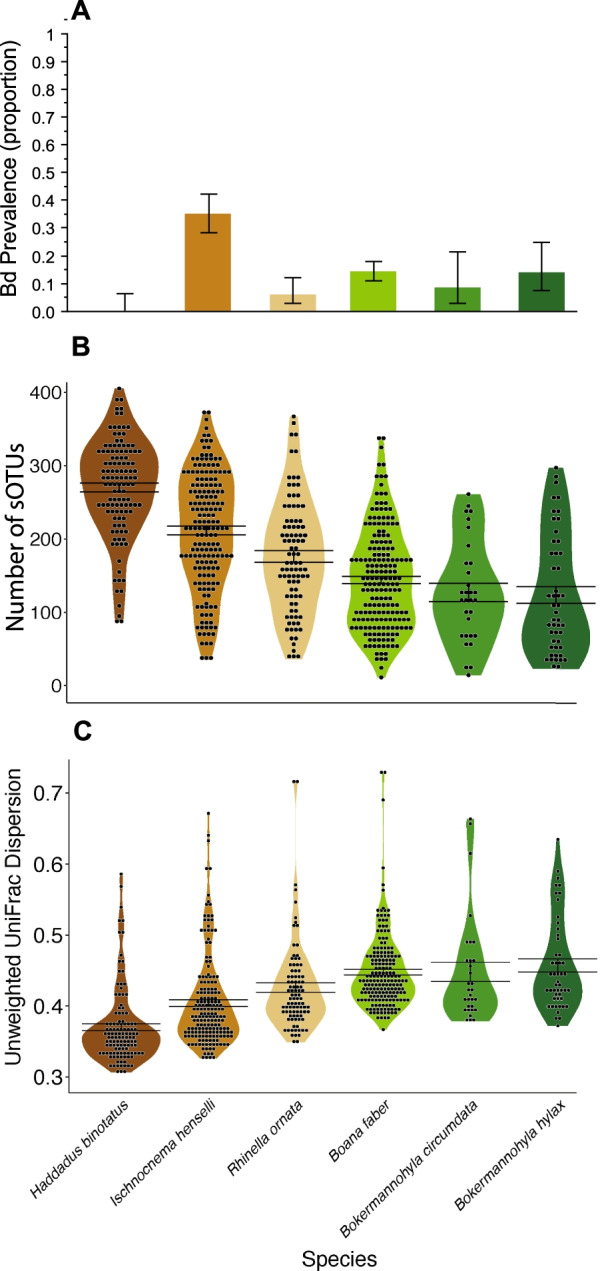
For six anuran species sampled in Brazil’s Atlantic Forest, **A** average prevalence of *Batrachochytrium dendrobatidis* (± standard error) across all samples, and violin plots showing likelihood of different **B** numbers of bacterial sub-operational taxonomic units (sOTU) detected on the skin (after rarefying sequence reads) and **C** levels of skin bacterial dispersion (distance to group centroid) based on unweighted UniFrac distances. Bars on violin plots indicate mean ± standard error

Bd prevalence varied significantly by species (X^2^ = 103.476, n = 903, *p* < 0.0001; Fig. 2A). We did not detect Bd in any of the 126 sampled *H. binotatus* individuals. Bd prevalence was 34.8% (n = 204) in *I. henselii*, 5.4% (n = 112) in *R. ornata*, 13.8% (n = 356) in *Bo. faber*, 7.0% (n = 43) in *Bk. circumdata*, and 12.7% (n = 63) in *Bk. hylax* (Fig. 2A). Prevalence patterns among the full dataset (n = 903) were similar to patterns for the subset of samples (n = 666) analyzed after rarefying bacteriome data to account for unequal sequencing depths among samples (X^2^ = 101.599, n = 666, *p* < 0.0001; Additional file 1: Table S1). *I. henselii* had an average (± standard error) Bd load of 246.9 ± 91.3 ITS copies, *R. ornata* 24.8 ± 15.5 ITS copies, *Bo. faber* 145.0 ± 46.0 ITS copies, *Bk. circumdata* 738.0 ± 612.9 ITS copies, and *Bk. hylax* 2,441.3 ± 2,379.7 ITS copies, but these averages did not differ significantly (F = 1.309_(4,132)_, R^2^ = 0.038; *p* = 0.270).

### Patterns of skin bacterial alpha diversity, composition and dispersion

Bacteriome alpha diversity metrics differed among host species: Shannon index (F_(5,548.7)_ = 36.12, R^2^ = 0.24, *p* < 0.0001), Faith’s phylogenetic diversity (F_(5,598.5)_ = 48.66, R^2^ = 0.30, *p* < 0.0001), and sOTU richness (F_(5,501.7)_ = 55.10, R^2^ = 0.32, *p* < 0.0001; Fig. 2B). *Haddadus binotatus* had higher bacteriome alpha diversity in all pairwise comparisons (Additional file 1: Table S2).

**Fig. 3:**
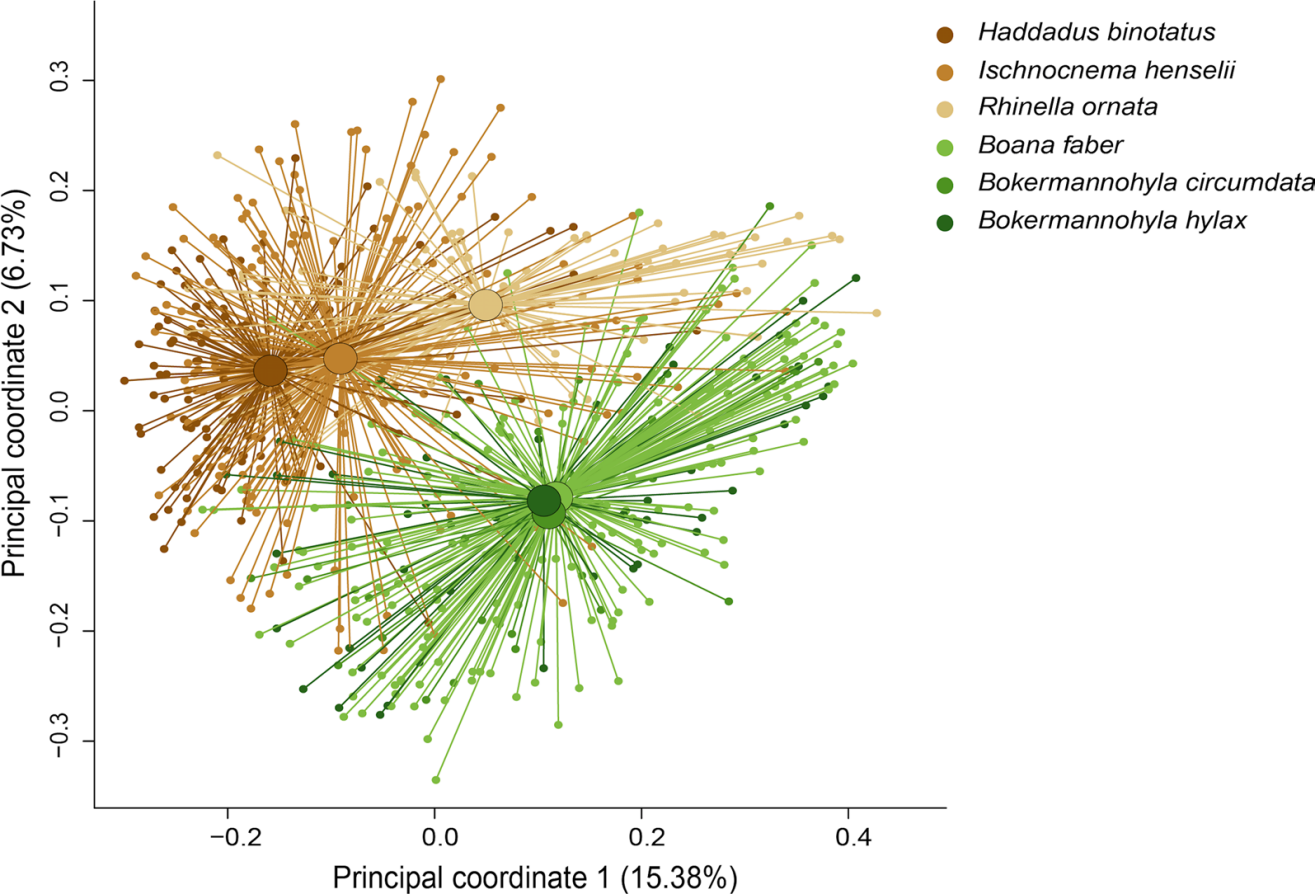
Community composition of host skin bacteria calculated using principal coordinates analysis based on unweighted UniFrac distances. Small circles indicate data points. Large circles indicate group centroids. Lines indicate distance to group centroids

Bacteriome beta diversity metrics also differed among species, both in terms of sOTU presence/absence (unweighted UniFrac PermANOVA: F_(4,660)_ = 16.82, R^2^ = 0.11, *p* = 0.001; Fig. 3) and relative abundance (weighted UniFrac PermANOVA: F_(4,660)_ = 49.89, R^2^ = 0.27, *p* = 0.001; Additional file 1: Fig. S1; Additional file 1: Table S3). The two terrestrial-breeding species and the toad species carried distinct skin communities while the three treefrog species hosted more similar communities (Fig. 3; Additional file 1: Table S3). Skin bacterial community composition did not differ between Bd-positive and Bd-negative *I. henselii* (unweighted UniFrac permANOVA: F_(1, 180)_ = 0.996, R^2^ = 0.006, *p* = 0.452; weighted UniFrac permANOVA: F_(1, 180)_ = 0.779, R^2^ = 0.004, *p* = 0.502).

Differential abundance analysis (Linear discriminant analysis Effect Size; LEfSe) revealed 87 differentially abundant sOTUs across the six landscapes where *H. binotatus* were sampled (Additional file 1: Fig. S2). Of these sOTUs, 65 (75%) were differentially abundant in *H. binotatus* in at least one landscape (Additional file 1: Fig. S2). *Haddadus binotatus* carried the highest number of differentially abundant sOTUs at all but one landscape where it was sampled (Additional file 1: Table S4). Four soil-associated sOTUs were enriched in *H. binotatus* at three landscapes, including *Devosia* sp. (Hyphomicrobiaceae), *Aminobacter* sp. (Phyllobacteriaceae), *Agrobacterium* sp. (Rhizobiaceae), and *Variovorax* sp. (Comamonadaceae; Additional file 1: Fig. S2; Additional file 1: Table S4).

Skin bacterial dispersion differed among host species (unweighted UniFrac: F_(5,122.1)_ = 33.02, R^2^ = 0.19, *p* < 0.0001; weighted UniFrac: F_(5,609.3)_ = 38.47, R^2^ = 0.24, *p* < 0.0001; Jaccard: F_(5, 553.8)_ = 56.42; R^2^ = 0.32; *p* < 0.0001; Bray–Curtis: F_(5, 652.6)_ = 40.76, R^2^ = 0.27, *p* < 0.0001; Additional file 1: Tables S5, S6). *Haddadus binotatus* had the lowest values of unweighted Unifrac (Fig. 2C) and Jaccard (Additional file 1: Fig. S3A) dispersion. Patterns of weighted UniFrac (Additional file 1: Fig. S3B) and Bray–Curtis (Additional file 1: Fig. S3C) dispersion were less consistent (Additional file 1: Tables S5, S6).

Skin bacterial communities across all six host species were dominated by taxa in the phyla Proteobacteria, Actinobacteria, and Bacteroidetes (Additional file 1: Fig. S4). More than half of detected sOTUs (58.9%) were shared by a proportion of individuals of all 6 host species and only 3.7% of sOTUs were unique to one host. *Haddadus binotatus* carried only two unique sOTUs (Additional file 1: Table S7) but hosted the highest diversity of common core (detected in at least 90% of individuals) sOTUs (12), compared to one common core sOTU for *I. henselii,* three common core sOTUs for *R. ornata*, two common core sOTUs for *Bk. hylax*, one common core sOTU for *Bk. circumdata*, and no common core sOTUs identified for *Bo. faber* (Additional file 1: Table S8). Most of the common core sOTUs for *H. binotatus* were also differentially abundant in this species (Additional file 1: Table S8). None of the common core sOTUs detected in *H. binotatus* were detected in the common core bacteriome of the other host species, except for an *Agrobacterium* sp. which was also detected in the common core bacteriome of *R. ornata* (Additional file 1: Table S8).

### Co-occurrence networks

To assess community interactions within host bacteriomes, we used co-occurrence networks based on significant pairwise correlations (both positive and negative) among sOTUs. Overall, bacteriome co-occurrence network topology in Serra do Japi, the best-sampled landscape, varied among *H. binotatus*, *I. henselii*, *R. ornata*, and *Bo. faber* (Table 1). To compare network topologies, we used a set of network metrics to characterize the interconnectedness (density, average path length, diameter, and average degree), clustering (average clustering coefficient and modularity), and centrality (betweenness centrality) of sOTUs within co-occurrence networks (Additional file 1: Methods). These comparisons revealed that observed networks were more interconnected (2- to fourfold higher diameters and average path lengths) and clustered (20- to 80-fold higher average clustering coefficients) than expected based on random networks. The exception to this pattern was the modularity metric (i.e., presence of correlated subsets [modules] of sOTUs), which was strikingly lower than expected for *I. henselii*, but four to six times higher than expected for the other host species. The proportion of positive correlations in the networks was high across all host species, ranging from 98% (*H. binotatus*) to 100% (*Bo. faber*). Average path length (a metric of network interconnectedness) was similar across all four host networks.

**Table 1:** Topology metrics of similarity-based bacterial networks using Spearman correlations (coefficient ρ > 0.6 and < − 0.6; *P* ≤ 0.01)

	Network	Edges	Nodes	Avg. clustering coefficient	Avg. shortest path length	Modularity	Graph density	Network diameter	Avg. degree	Bd Prevalence (%)	Avg. sOTU richness
*H. binotatus*	Observed	3555	724	0.670	5.210	0.670	0.014	15	9.820	0	247 ± 62
	Random			0.014 ± 0.001	3.130 ± 0.003	0.241 ± 0.007	–	5.162 ± 0.369	–		
*R. ornata*	Observed	13,107	1000	0.493	4.093	0.59	0.026	15	26.214	3.8	179 ± 85
	Random			0.026 ± 0.00	2.463 ± 0.001	0.128 ± 0.003	–	3.969 ± 0.173	–		
*B. faber*	Observed	5654	881	0.575	5.657	0.809	0.015	15	12.835	29.2	162 ± 70
	Random			0.015 ± 0.001	2.915 ± 0.002	0.203 ± 0.005	–	4.968 ± 0.176	–		
*I. henselli*	Observed	6146	971	0.45	4.589	0.029	0.013	12	12.659	37.2	202 ± 72
	Random			0.013 ± 0.001	2.967 ± 0.001	0.202 ± 0.005	–	4.999 ± 0.032	–		

Network metrics for each amphibian species represent those from the observed and respective Erdös-Réyni random networks. Networks include samples from the Serra do Japi landscape only. Bd prevalence and bacterial sOTU richness for each host species are those estimated for Serra do Japi

**Fig. 4:**
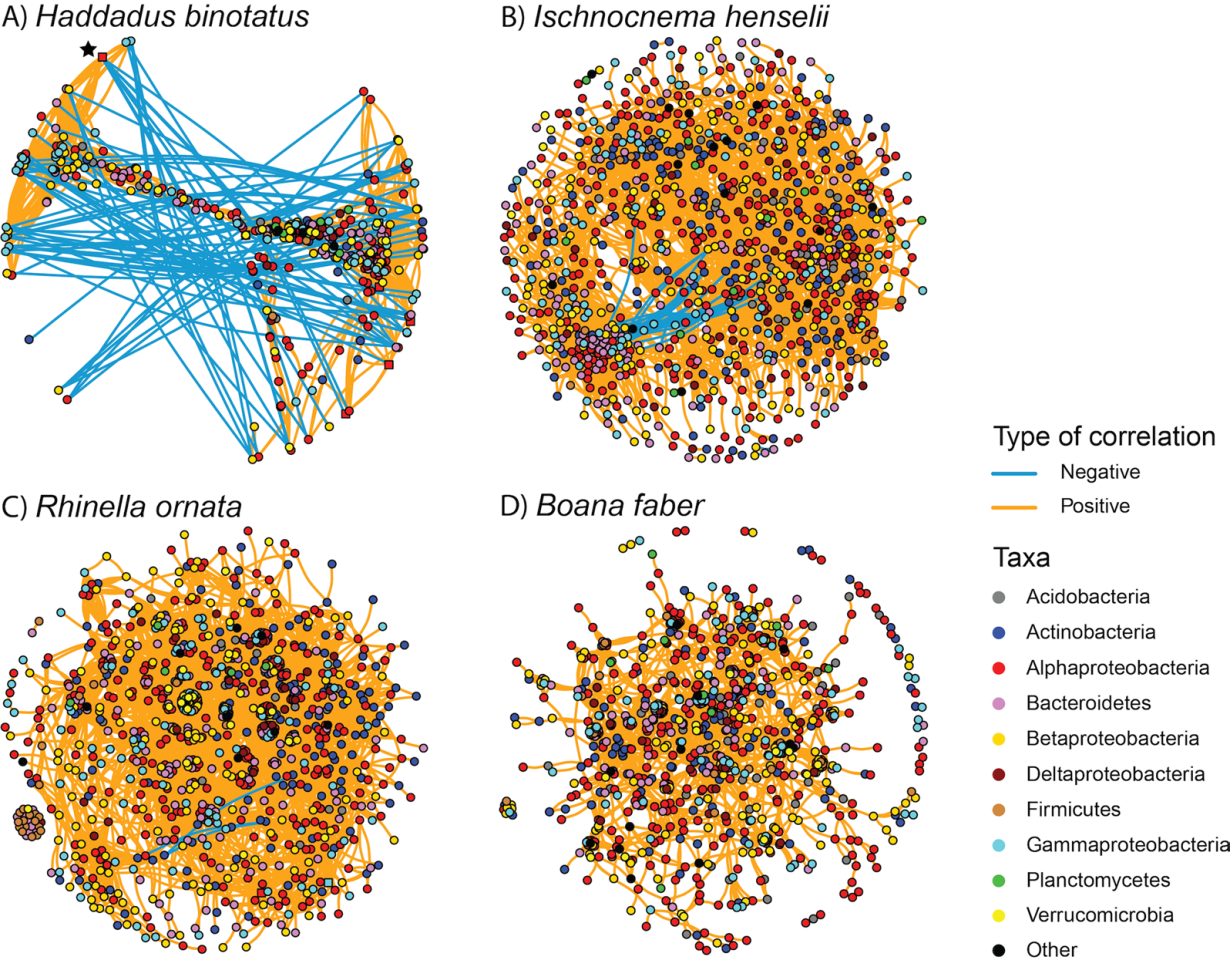
Correlation-based bacterial networks for the Serra do Japi landscape using Spearman correlations (coefficient ρ > 0.6 and < − 0.6; *p* ≤ 0.01) and the Fruchterman-Reingold layout. Colored points indicate nodes (sOTUs) and lines indicate edges (positive or negative correlations). Nodes (sOTUs) are color-coded based on bacterial phylum or class (in the case of Proteobacteria). Square-shaped nodes represent common core sOTUs (90%). In the *Haddadus binotatus* network, the common core sOTU classified as Alphaproteobacteria and shown at the top left of the network (marked with a black star) ranked highest in betweenness centrality. Edge lengths are a function of layout and are not biologically meaningful

*Haddadus binotatus*, the species harboring no detectable Bd, had the smallest bacterial network in number of nodes (sOTUs) and edges (correlations). This network was relatively sparsely connected (based on average degree), and highly clustered (based on average clustering coefficient and modularity; Table 1). This network was partitioned into two main modules connected by a subset of nodes and primarily separated by negative edges, a distinct clustering pattern not observed in the other host species (Fig. 4A).

In *I. henselii*, the species that harbored the highest Bd prevalence, network topology ranked low in measures of both overall interconnectedness and clustering. This network showed the lowest diameter, average clustering coefficient and modularity (Table 1), visualized as one indistinct cluster of nodes (Fig. 4B). *R. ornata* had the largest and most interconnected network with the highest average degree and density, but intermediate clustering (Fig. 4C; Table 1). The *Bo. faber* network ranked highest in modularity, though it differed from *H. binotatus* in the lack of negative edges and high number of small modules at the network periphery (Fig. 4D; Table 1).

Of the 12 common core sOTUs detected in *H. binotatus*, 9 sOTUs were included in its network, one of which (*Paracoccus marcusli* from the class Alphaproteobacteria) presented the highest betweenness centrality value (i.e., degree to which the positioning of an sOTU bridges different parts of the network) in the entire network (and 22°, Fig. 4A). Most common core sOTUs for *I. henselii* and *R. ornata* were included in their networks but did not present high centrality values.

## Discussion

We conducted the first comparative bacteriome study of terrestrial and aquatic-breeding frogs in Brazil’s Atlantic Forest, with high spatial replication (40 sampling sites across eight landscapes) and robust landscape-level spatial coverage (400 km). Strikingly, Bd was not detected in more than 100 sampled individuals of the terrestrial-breeding species *H. binotatus.* In contrast, we detected 35% Bd prevalence among 204 individuals of the ecologically similar, terrestrial-breeding species *I. henselii*, 7–14% Bd prevalence among 462 individuals of three aquatic-breeding treefrog species, and 5% Bd prevalence among 112 individuals of the aquatic-breeding toad *Rhinella ornata* (Fig. 2A). Previous work on Bd dynamics in Brazilian terrestrial-breeding frogs suggests low Bd resistance in these species [49] and substantial exposure to Bd in the wild [44, 54]. For example, *Brachycephalus ephippium*, another terrestrial-breeding frog species endemic to Brazil’s Atlantic Forest with similar habitat use as our focal terrestrial species, was exposed to substantial levels of Bd in terrestrial habitats through spillover from aquatic-breeding species during the breeding season [48]. Thus, the contrasting Bd occurrence patterns we observed between *H. binotatus* and *I. henselii* may be more strongly driven by host factors than environmental or pathogen factors, prompting a comparative skin bacteriome analysis of an ecologically diverse assemblage of amphibian species, some for the first time.

In contrast to consistent non-detections of Bd in *H. binotatus*, the highest Bd prevalence in our study was found in *I. henselii*, the only other species in our study with a terrestrial-breeding life history strategy, and which can be found side by side with *H. binotatus* in the field. While the skin bacteriomes of Atlantic Forest treefrog species tracked bioclimatic variables at large spatial scales [55], the finer spatial resolution of our study revealed significant species-level variation in host bacteriome attributes under constant biogeographic and climatic conditions, consistent with previous work showing differences in the skin bacterial communities of co-occurring amphibian species [56–59]. Compared to *I. henselii*, *H. binotatus* carried significantly higher overall bacterial alpha diversity (Fig. 2B). High alpha diversity could enhance Bd inhibition if diverse microbial communities have a greater chance of containing Bd-suppressing taxa and these taxa are able to become dominant [50, 60]. However, *I. henselii* carried the second highest bacteriome alpha diversity of the six species evaluated (Fig. 2B), suggesting that overall bacterial diversity on its own does not explain differential Bd prevalence among the focal host assemblage. Similarly, *H. binotatus* scored the lowest in relative bacterial dispersion but *I. henselii* had the second lowest dispersion (Fig. 2C), similarly suggesting that the dispersion metric alone does not fully explain host-Bd interactions in this system.

The most contrasting bacteriome characteristics between *H. binotatus* and *I. henselii* were common core diversity and co-occurrence network structure. While we detected only one common core sOTU in *I. henselii*, the common core bacteriome of *H. binotatus* contained 12 sOTUs. Species richness of common core microbial communities, defined as the taxa occurring above an occupancy frequency threshold, is not necessarily linked to bacteriome function or evolutionary history with the host [61]. Certain taxa can have high colonization frequency within host populations for many reasons, including high competitive ability or widespread distribution in the environment, and certain taxa may play important functional roles in host populations even when rare [61]. As a result of these findings, recent work has differentiated the common core from other types of core microbiomes including the temporal core, ecological core, functional core, and host-adapted core [61]. Co-occurrence network analysis uses centrality metrics to identify sOTUs that may play an important role in structuring the microbiome, paralleling the concept of the ecological core [61–63]. Aside from high relative diversity, the common core bacteriome of *H. binotatus* included the sOTU with the highest betweenness centrality score in the co-occurrence network for this species (black star in Fig. 4A), suggesting a highly connected position that could stabilize and regulate community structure and occurrence of taxa with beneficial attributes [62, 63]. Our results support recent perspectives on core microbial communities as good candidates from which to build on our understanding of host-microbe interactions and bacteriome function [61].

A conspicuous attribute of the *I. henselii* network was low modularity, both compared to other observed host networks and randomly generated networks (Fig. 4B). In contrast, the *H. binotatus* network had a highly clustered structure with two main modules joined by a subset of sOTUs and distinct partitioning between positive and negative sOTU frequency correlations (Fig. 4A). This partitioning is unlikely to be a geographical artifact because our bacteriome networks represent frogs from a single landscape. Network modularity reveals clusters of associated species that cohabitate, potentially indicating niche partitioning mediated by competition, mutualism, or host microhabitat characteristics [53, 64]. In contrast, the central nodes joining the two modules could be more generalist taxa. Across different types of ecological networks, modularity has been associated with network function and robustness [64–68]. For instance, high modularity could increase efficiency in sharing of metabolites across the network, reduce ripple effects of network perturbations, or promote functional redundancy to guard against failure of certain metabolic reactions [69, 70]. Our findings suggest a high level of network organization in *H. binotatus*, which could indicate functional communities with patterns of assembly and ecological interactions that happen to be effective against Bd invasion, even under the probable scenario that Bd is not a prominent selective force on bacteriome structure in terrestrially associated frogs [9, 21, 53].

Specifically, the highly structured negative correlations among sOTUs in the *H. binotatus* network (blue lines in Fig. 4A) could indicate a bacteriome primed for antagonistic interactions and thus more resilient to Bd invasion [52, 71], consistent with the finding that modules of marine sponge bacteriomes were associated with production of bioactive secondary metabolites involved in chemical defense [70]. Similar to *H. binotatus*, Bd prevalence is typically low or absent in North American eastern red-backed salamanders *Plethodon cinereus*, a species that carries high concentrations of violacein, a strongly antifungal secondary metabolite of the dominant skin bacterium *Janthinobacterium lividum* [72–77]; thus, a comparison of modularity in the bacteriomes of these two species would be useful. The *H. binotatus* network also stood apart in its relative sparseness with the fewest nodes, edges, and edges per node, which contrasts with the high overall taxonomic diversity of the bacteriome. Together, these results suggest that high overall bacteriome alpha diversity could function as a rich bacterial reservoir facilitating the formation of a highly structured and synergistic subset of member taxa, consistent with the finding that synergistic interactions among diverse microbial assemblages could boost the Bd-inhibitory functions of the community [50].

Compared to the focal terrestrial-breeding species, the focal aquatic-breeding treefrog species carried intermediate Bd prevalence, low overall bacteriome alpha diversity, high bacterial dispersion, and scant to absent common cores (0–2 sOTUs). The most distinctive feature of the *Bo. faber* co-occurrence network was a high number of small node clusters loosely connected to the overall network. This finding suggests a substantial level of bacterial community variability among individuals, consistent with variable microhabitat use and potentially helping to explain the high dispersion and few common core microbes characteristic of the generalist treefrog species. The *Bo. faber* network also lacked negative connections, suggesting limited antagonistic activity, such as production of anti-microbial metabolites that could protect against microbial invasion [76].

The focal aquatic-breeding toad *R. ornata* carried relatively low Bd prevalence consistent with previous surveillance [54], intermediate bacteriome alpha diversity and dispersion, and three common core sOTUs. The *R. ornata* network was highly interconnected, scoring twice as high as other species in overall network density and average number of connections per sOTU, suggesting an organized structure with complex paths of communication. Thus, acknowledging the limitations of our correlational results and the vast life history differences among our focal host species, one consistency across life history guilds was lower Bd prevalence in species with higher bacteriome network structure (*H. binotatus* and *R. ornata*) and higher Bd prevalence in species with less defined network structure (*I. henselii* and *Bo. faber*). This pattern is consistent with the previous finding, in a comparison of three Panamanian amphibian species, that the more Bd-resistant species had more bacteriome network connections and greater clustering than the more Bd-vulnerable species [78]. Similarly, the bacteriome of Bd-vulnerable species were more similar to environmental bacterial pools compared to Bd-resistant species [58], suggesting that skin communities formed mainly by neutral processes may be less protective than more structured communities formed by deterministic processes.

While the distinctive skin bacteriome of *H. binotatus* is compelling as a mechanism explaining the lack of Bd detected in this species, not all bacteriome metrics that we measured were correlated with Bd infection (e.g., sOTU richness, dispersion) and, thus, it is critical to consider other possible non-mutually exclusive factors that could contribute to this pattern. Studies on the physiology of *H. binotatus* are extremely scarce and thus we have limited ability to assess skin structure and other physiological factors that could contribute to Bd resistance, but distinctive characteristics of the skin such as sloughing rate have been associated with Bd resistance in other species [79]. Behavioral studies are also rare. Both *I. henselii* and *H. binotatus* were observed in forested areas near and away from streams. During dryer months, *I. henselii* may be observed very close to streams and occasionally submerged in water [80], whereas *H. binotatus* has been observed in holes and under debris during dryer periods in other studies, possibly because they are larger-bodied and more tolerant of dry conditions [81]. These divergent strategies for overcoming hydric stress may lead to different encounter rates with environmental Bd reservoirs (streams) and environmental bacterial reservoirs. However, our study took place in the rainy season when frogs were unlikely to be experiencing hydric stress and both terrestrial-breeding species were likely exposed to similar Bd and bacterial reservoirs within the same terrestrial microhabitats.

The bacteriome is only a portion of host-associated microbial communities, with protists, multicellular fungi, viruses, archaea, and microscopic metazoans also often in great abundances [82–85]. Microbial assemblages also exist in a complex mucosal substrate that includes host-produced antimicrobial peptides [86, 87]. These additional components of host skin communities interact with host-associated bacteria [84, 85] and may also independently interact with invading microbes [86, 87], suggesting that analysis at the level of the entire chemical and biological mucosal matrix may be necessary to fully evaluate Bd resistance mechanisms in *H. binotatus*. Immunogenetic variation is another factor that could not only help to explain host variation in microbiome assembly [85, 88, 89], but could also contribute to Bd resistance independently of the microbiome [37, 38, 90, 91]. Thus, immunogenetic analysis of our focal species could reveal a more complete mechanistic picture of host variation in Bd resistance.

We cannot rule out the possibility that *H. binotatus* possesses low resistance and tolerance to Bd and thus succumbs to chytridiomycosis extremely quickly, to an extent that lowers the chances of detecting infected individuals. This scenario seems unlikely, however, because we would expect to find a small number of individuals with very high infection loads over the course of our intensive sampling effort. Variation in landscape characteristics is also unlikely to explain the absence of detectable Bd in our *H. binotatus* samples because the pattern was consistent across all six landscapes where *H. binotatus* were found. Similarly, variation in host reproductive strategy and phylogenetic classification are unlikely explanations because *H. binotatus* and *I. henselii* are phylogenetically similar, both belonging to the Brachycephaloidea superfamily, and both exhibit direct development. *I. henselii* typically exhibit higher population densities than *H. binotatus*, which could increase disease transfer between conspecifics. However, *H. binotatus* were also generally abundant at our sample sites, so this probably does not explain the lack of Bd found in *H. binotatus*. *Haddadus binotatus* and *I. henselii* generally share the same forested habitats and activity patterns. However, we observed *I. henselii* vocalizing on the ground as well as within bromeliads and on leaves, branches, and trunks up to 1.5 m above the ground, consistent with the few previous natural history observations for the species [80]. In contrast, we never observed *H. binotatus* in bromeliads or vocalizing at heights greater than 20 cm from the ground, although they have occasionally been observed up to 80 cm above the ground [92]. Additional research is needed to characterize the habitat use of these poorly studied species more thoroughly, but if *H. binotatus* and *I. henselii* come in contact with certain substrates at different rates, this could lead to different patterns of bacterial recruitment.

## Conclusion

Direct-developing frogs have been found to be at high risk of population declines caused by climate change and chytridiomycosis [28, 40, 49, 93]; however, our study has shown evidence of Bd resistance in the direct-developing species *H. binotatus*. The bacteriomes of *H. binotatus* were highly diverse and structured, most notably with high common core taxonomic diversity and a tightly clustered network topology partitioned by negative correlations, characteristics that may be associated with community stability and competitive interactions that could inhibit Bd colonization. Together, our results indicate that bacteriome community structure could be contributing to Bd resistance in *H. binotatus*, although further research is needed to rule out other resistance factors, especially because our study represents the first analysis of the *H. binotatus* microbiome and natural history information on this species is scarce. Continued research on the mechanisms that prevent infection in *H. binotatus* may prove to be beneficial in conservation efforts to protect diverse radiations of cryptic direct-developing species from Bd-induced population declines and extinctions. This includes culturing and challenging *H. binotatus* skin bacteria with Bd to determine the influence of key taxa, or networks of taxa, on Bd resistance; studies on behavioral, genetic, and mucosomal drivers of host-associated bacterial composition; genome-resolved metagenomics and metatranscriptomics to elucidate functional roles of the bacteriome in Bd resistance [94]; and manipulation experiments to detect keystone bacteriome members and probiotic assemblages.

## Methods

### Study location and focal species

We sampled eight landscapes (circular areas 15 km in diameter) in the Atlantic Forest in the state of São Paulo, Brazil (Fig. 1). Within each landscape, we sampled five natural forest sites (Fig. 1). We conducted field sampling during the breeding season from September 2018 to January 2019, visiting each of the 40 sites twice within that time.

We sampled six common species (Fig. 1) comprising two modes of reproduction. Four species are aquatic-breeding, including three arboreal treefrog species, *Boana faber*, *Bokermannohyla circumdata*, and *Bk*. *hylax*, and the toad *Rhinella ornata*. Two species, *Haddadus binotatus* and *Ischnocnema henselii*, are terrestrial-breeding. All of these species are endemic to the Brazilian Atlantic Forest [43]. Frogs were captured primarily in terrestrial leaf litter and vegetation, rarely within water bodies.

Across all sites, we captured 903 individual frogs: 356 *Bo*. *faber*, 42 *Bk*. *circumdata*, 63 *Bk*. *hylax*, 126 *H*. *binotatus*, 204 *I*. *henselii*, and 112 *R*. *ornata* (Additional file 1: Table S1). We captured each individual frog with fresh gloves, rinsed the skin surface with sterile distilled water to remove transient bacteria, and collected skin swabs following Hyatt et al. [95]. We stored swabs in sterile tubes on ice in the field and transferred samples to -20 ºC until DNA extraction (Qiagen DNeasy; Additional file 1: Methods). After swabbing, we released all individuals at the point of capture.

### Bd and bacteriome characterization

To quantify Bd loads, we followed qPCR protocols by Boyle et al. [96]. For qPCRs, we diluted an aliquot of each extracted DNA sample 1:10 and used primers to amplify the ITS and 5.8S rRNA regions of Bd, including synthetic standards ranging from 10^2^ to 10^6^ gene copies [96].

To characterize frog skin bacterial communities, we followed the Earth Microbiome Project 16S Illumina Amplicon Protocol, targeting the V4 region of the bacterial 16S rRNA gene and including negative controls without template DNA (Additional file 1: Methods) [97, 98]. We used Quantitative Insights into Microbial Ecology (QIIME 2) v. 2019.1 for initial processing of bacterial sequences (Additional file 1: Methods). We clustered amplicon sequences into sub-operational taxonomic units (sOTUs) using Deblur [99], discarded sOTUs with fewer than 0.005% of the total sequence reads in the dataset [100] and rarefied samples to 2,000 reads to standardize read counts across samples (Additional file 1: Methods).

For analyses of alpha diversity, we calculated sOTU richness, Shannon index, and Faith’s phylogenetic diversity for each sample. We also identified common core sOTUs for each host species, defined as the set of sOTUs detected in at least 90% of sampled individuals, independent of relative abundance.

For analyses of beta diversity, we calculated Jaccard, Bray–Curtis, and unweighted and weighted UniFrac distances between samples [101, 102]. To quantify bacterial dysbiosis, referring to imbalance that may disrupt healthy functioning, we used the metric known as dispersion. In many cases, dysbiosis occurs through stochastic changes in the microbiome, leading to increased microbial variability among hosts [103]. Thus, dispersion, a measure of microbial variability, is routinely used as a measure of dysbiosis [103–105]. We used Jaccard, Bray–Curtis, and unweighted and weighted UniFrac distances between samples to calculate bacterial dispersion for each sample as an index of dysbiosis (betadisper function in vegan package in Program R). Dispersion is estimated by reducing original distances to principal coordinates and calculating the non-Euclidean distance between each object and the group centroid. We used amphibian species as the grouping variable.

### Statistical and network analyses

We compared infection prevalence among amphibian species using a Generalized Linear Model (GLM) with binomial distribution and logit link. We used General Linear Mixed Models (GLMMs; standard least squares) to compare bacterial alpha diversity indices and bacteriome dispersion among amphibian species. Study landscape (1–8) and site (1–5) within landscape were included as random effects. We performed Tukey a posteriori tests for each pairwise comparison. We compared average Bd infection loads (log transformed; only Bd-positive individuals) among amphibian species using a similar GLM approach. We used permutational analysis of variance (permANOVA) to compare skin bacterial composition among species (Additional file 1: Methods). To test for effects of Bd infection status on microbiome composition, we used permANOVAs to compare skin bacterial composition (weighted and unweighted UniFrac distance) between Bd-positive (n = 66) and Bd-negative (n = 116) *I. henselii*, the focal species with the most robust sample sizes of Bd-positive and Bd-negative individuals. To identify differentially abundant sOTUs among host species for each study landscape, we used the linear discriminant analysis (LDA) effect size (LEfSe) method [106](Additional file 1: Methods).

To compare patterns of sOTU co-occurrence among host skin bacterial communities, we computed correlation-based networks (Additional file 1: Methods). In these networks, each node represents an sOTU and edges between nodes represent highly significant pairwise correlations, which can be inferred as co-occurrences (when ρ is positive) or antagonistic interactions (when ρ is negative). We characterized network structure from the following metrics: density, average shortest path length, diameter, average degree, average clustering coefficient, modularity, and node betweenness centrality (Additional file 1: Methods). We limited this analysis to a single landscape to reduce effects of confounding factors associated with variation between landscapes. Thus, we selected Serra do Japi, the landscape with the largest sample size (n = 120 individuals) of the eight focal landscapes. Sample sizes at this landscape were well-balanced between terrestrial-breeding (n = 21 *H. binotatus*, n = 43 *I. henselii*) and aquatic-breeding (n = 24 *Bo. faber*, n = 26 *R. ornata*) host species. *Bk. hylax* (n = 5) and *Bk. circumdata* (n = 1) were rarely sampled at this landscape and were excluded. Bacteriome diversity and Bd infection prevalence at this landscape followed trends for the eight landscapes combined (Table 1; Additional file 1: Table S1).

## Supplementary Information

**Additional file 1:** Supplementary methods, tables and figures.

## Data Availability

The datasets generated and analysed during the current study are available from the corresponding authors on reasonable request.

## Abbreviations

Bd: *Batrachochytrium dendrobatidis*
GLM: Generalized Linear Model
GLMM: General Linear Mixed Models
LEfSe: Linear discriminant analysis (LDA) effect size
permANOVA: Permutation analysis of variance
QIIME: Quantitative Insights into Microbial Ecology
sOTU: Sub-operational taxonomic unit
