# Supplementary material for: Signatures of functional bacteriome structure in a tropical direct-developing amphibian species
Source: Anim Microbiome. 2022 Jun 7;4:40. doi: 10.1186/s42523-022-00188-7 (PMC9172097; doi:10.1186/s42523-022-00188-7)
Supplement: Supplementary file 1 — Additional file 1: Supplementary methods, tables and figures. [file 42523_2022_188_MOESM1_ESM.docx]

**Additional file 1**

**Signatures of functional bacteriome structure in a tropical direct-developing amphibian species**

Renato A. Martins^a,^*, Sasha E. Greenspan^b,^*, Daniel Medina^c^, Shannon Buttimer^b^, Vanessa M. Marshall^b^, Wesley J. Neely^b^, Samantha Siomko^b^, Mariana L. Lyra^d^, Célio F. B. Haddad^d^, Vinicius Sao Pedro^e^, C. Guilherme Becker^b,f^

^a^Programa de Pós-graduação em Conservação da Fauna, Universidade Federal de São Carlos, São Carlos, SP 13565-905, Brazil

^b^Department of Biological Sciences, The University of Alabama, Tuscaloosa, AL 35487, USA

^c﻿^Sistema Nacional de Investigación, SENACYT, Building 205, City of Knowledge, Clayton, Panama, Republic of Panama

^d﻿^Department of Biodiversity and Aquaculture Center (CAUNESP), Universidade Estadual Paulista, Rio Claro, SP 13506-900, Brazil

^e^Centro de Ciências da Natureza, Universidade Federal de São Carlos, Campus Lagoa do Sino, Buri, SP 18290-000, Brazil

^f^Department of Biology, The Pennsylvania State University; University Park, PA 16803, USA

*These authors contributed equally to this work

Corresponding author emails: Renato A. Martins: diarioanimaloficial@gmail.com, Sasha E. Greenspan: sasha.greenspan@gmail.com

Supplementary methods

*Molecular procedures*

We performed DNA extractions using the Qiagen DNeasy kit (Qiagen), following the manufacturer’s protocol with the exception of extending the 56 ºC incubation period to overnight to increase DNA yield. We diluted an aliquot of each extracted DNA sample 1:10 for qPCR analysis of Bd infection loads. We used primers to amplify the ITS and 5.8S rRNA regions of Bd and measured loads using synthetic standards ranging from 10^2^ to 10^6^ gene copies [1].

To characterize frog skin bacterial communities, we followed the Earth Microbiome Project 16S Illumina Amplicon Protocol, targeting the V4 region of the 16S rRNA gene using universal primers 515F and 806R and a dual index strategy [2–4]. We amplified extracted DNA in duplicate using Phire Hot Start II DNA Polymerase (Finnzyne, Espoo, Finland), including a negative control without template DNA on each 96-well plate. We then visualized amplicons in 1% agarose gel to confirm consistent gel band strength and pooled samples in equal volumes to generate the amplicon library. We purified the library using the QIAquick Gel Extraction Kit (Qiagen, Valencia, CA, USA) and measured DNA concentration of the purified sample using the Qubit 2.0 fluorometer with the dsDNA Broad-Range Assay Kit (ThermoFisher Scientific, San Jose, CA, USA). The library was sequenced on an Illumina MiSeq (2 x 250 bp) sequencer at the Tufts University Core Facility (TUCF Genomics), Boston, Massachusetts, USA.

*Bioinformatics*

We used Quantitative Insights into Microbial Ecology (QIIME 2) v. 2019.1 for initial processing of bacterial sequences[5]. We used the q-score plugin to filter low-quality reads and the deblur workflow [6] to cluster sequences into sub-operational taxonomic units (sOTUs). We used only forward reads for compatibility with the deblur [6] workflow and trimmed reads to 150 bp to maximize read quality. We aligned sequences (alignment plugin) with MAFFT [7] and constructed a phylogenetic tree (phylogeny plugin) using FastTree with QIIME default parameters [8]. We assigned taxonomy (feature-classifier plugin) with the scikit-learn naïve Bayes taxonomy classifier and the Greengenes 13.8 reference sequence database [9–11].

We discarded sequences identified as chloroplasts or mitochondria and eight sOTUs that were differentially abundant in the PCR controls compared to the samples, appearing to be contaminants. Seven of the contaminant sOTUs were assigned to the phylum Proteobacteria, including the families Bradyrhizobiaceae, Enterobacteriaceae, Caulobacteraceae, Rhodobacteraceae, Legionellaceae, Sphingomonadaceae, and one unidentified family. One of the contaminants was an unidentified OTU from the phylum Bacteroidetes. We also discarded sOTUs with fewer than 0.005% (438 sequence reads) of the 8,767,188 total sequence reads in the dataset [12]. To standardize read counts across samples, we rarefied samples to 2,000 reads. After all filtering steps, our final sOTU table consisted of 666 samples, including 1448 sOTUs and 1,344,000 sequence reads (Supplementary Table 1).

To quantify microbiome dysbiosis, referring to imbalance in the microbiome that disrupts its healthy functioning, we used the metric known as dispersion. In many cases, dysbiosis occurs through stochastic changes in the microbiome, leading to increased microbial variability among hosts [13]. Thus, dispersion, a measure of microbial variability, is routinely used as a measure of dysbiosis [13–15]. We used Jaccard, Bray-Curtis, and unweighted and weighted UniFrac distances between samples to calculate bacterial dispersion for each sample as an index of dysbiosis (betadisper function in vegan package in Program R). Dispersion is estimated by reducing original distances to principal coordinates and calculating the non-Euclidean distance between each object and the group centroid. We used amphibian species as the grouping variable.

We did not perform analyses using the database of bacteria with *Bd*-inhibitory and facilitating properties compiled by Woodhams et al. [16]. This database was formed using bacterial isolates from aquatic-breeding frogs from North and Central America. As we are studying South American terrestrial-breeding frogs this database would not be very informative.

*Statistical and network analyses*

We employed permutational analysis of variance (PermANOVA), based on unweighted and weighted UniFrac distance matrices, to compare skin bacterial composition among species using the adonis function from the vegan package in Program R [17,18]. To visualize differences in bacterial community composition, we performed principal coordinates analysis on UniFrac distances in QIIME 2 and plotted values for the first two principal coordinate axes with centroids using the pca2d function from the phytools package in Program R [19].

To identify differentially abundant sOTUs among host species for each study landscape, we used the linear discriminant analysis (LDA) effect size (LEfSe) method on the galaxy platform using default parameters [20,21]. We consolidated LefSE information and visualized the results on a single heat map using the heatmap.2 function in R (Heatplus package, R version 4.0.2). We identified sOTUs that were shared among species as well as sOTUs that were unique to certain species by constructing a Venn diagram in JMP [22]. We quantified common core sOTU diversity among species and identified *H. binotatu*s common core sOTUs that were also present in the LefSE differential abundance database.

Co-occurrence networks were calculated for each host species using pairwise Spearman correlations in Program R, and only highly significant correlations (*p* ≤ 0.01) with coefficients ρ above 0.6 and below -0.6 were included [23]. We calculated correlation and significance matrices using the function rcorr from the package Hmisc [24]. We adjusted P-values to control for false discovery rate using the function p.adjust following the Benjamini and Hochberg method [25]. To visualize networks, we used the Fruchterman-Reingold layout using the package igraph [26].

To make inferences about potential community properties, we calculated a set of metrics to characterize the structure of each host network. One subset of metrics characterized the overall interconnectedness of host networks, including density (number of edges out of total potential number of edges), average shortest path length (average shortest distance in number of edges between all pairs of nodes), diameter (number of edges of the longest geodesic path between any two nodes), and average degree (average number of edges connected to individual nodes). Another subset of metrics characterized the degree of aggregation within the network. One metric of aggregation is clustering, with a cluster referring to nodes and edges forming a triangle and, with average clustering coefficient referring to the number of observed triangles out of the total possible triangles for each node (average number of edges between neighbors of a node out of total potential number of edges between neighbors). Another metric of aggregation is modularity, (the degree to which correlations are confined to subsets of nodes, or ‘modules’, which ranges between 0, if all sOTUs interact equally, and 1, if OTUs only interact within modules).

Assessing network position of individual nodes can be used to identify sOTUs that may be important for the structure or stability of the network. For each sOTU previously identified as common core, we extracted ranked betweenness centrality. Betweenness centrality refers to the number of times a node lies on the shortest path between other nodes. A high betweenness centrality score indicates that an sOTU bridges different parts of the network and thus may play an important role in network cohesion, stabilization, and indirect interactions [27,28]. In addition, high centrality may be used to identify hub nodes, which have been associated with the concept of ‘keystone’ taxa, and whose removal from the network may have disproportionately deleterious effects on network structure and function due to their role in mediating ecological interactions [27].

To assess whether observed networks differed from random patterns of network topology expectations based on random network, we generated 1000 random networks for each species with numbers of nodes and edges matching each observed network using the Erdös–Réyni model [29], in which every possible edge between sOTUs has an equal probability of occurring. Random networks were computed using the erdos.renyi.game function (with argument ‘type’ = “gnm”) from the igraph package. Estimates of topology metrics of random networks represent the average and standard deviation of the 1000 networks generated. Observed and expected network density and average degree metrics were equal between observed and expected values because these metrics are determined by the number of nodes and edges in each network.

*References*

1. Boyle DG, Olsen V, Morgan JAT, Hyatt AD. Rapid quantitative detection of chytridiomycosis (*Batrachochytrium dendrobatidis*) in amphibian samples using real-time Taqman PCR assay. Dis Aquat Organ. 2004;60:141–8.

2. Caporaso JG, Kuczynski J, Stombaugh J, Bittinger K, Bushman FD, Costello EK, et al. QIIME allows analysis of high-throughput community sequencing data. Nat Methods [Internet]. 2010;7:335–6. Available from: http://link.springer.com/10.1007/978-1-4939-0897-4%0Ahttp://dx.doi.org/10.1038/nrgastro.2012.156%0Ahttp://dx.doi.org/10.1038/nature07540%0Ahttp://dx.doi.org/10.1038/srep34712%0Ahttp://www.nature.com/doifinder/10.1038/nbt.2676

3. Caporaso JG, Lauber CL, Walters WA, Berg-Lyons D, Huntley J, Fierer N, et al. Ultra-high-throughput microbial community analysis on the Illumina HiSeq and MiSeq platforms. ISME J [Internet]. Nature Publishing Group; 2012;6:1621–4. Available from: http://dx.doi.org/10.1038/ismej.2012.8

4. Kozich JJ, Westcott SL, Baxter NT, Highlander SK, Schloss PD. Development of a dual-index sequencing strategy and curation pipeline for analyzing amplicon sequence data on the MiSeq Illumina sequencing platform. Appl Environ Microbiol. 2013;79:5112–20.

5. Bolyen E, Rideout JR, Dillon MR, Bokulich NA, Abnet CC, Al-Ghalith GA, et al. Reproducible, interactive, scalable and extensible microbiome data science using QIIME 2. Nat Biotechnol [Internet]. 2019;37:848–57. Available from: http://www.nature.com/articles/s41587-019-0209-9

6. Amir A, McDonald D, Navas-Molina JA, Kopylova E, Morton JT, Xu ZZ, et al. Deblur rapidly resolves single-nucleotide community sequence patterns. mSystems. 2017;2:e00191-16.

7. Katoh K, Misawa K, Kuma K, Miyata T. MAFFT: a novel method for rapid multiple sequence alignment based on fast Fourier transform. Nucleic Acid. 2002;30:3059–66.

8. Price MN, Dehal PS, Arkin AP. FastTree 2 - approximately maximum-likelihood trees for large alignments. PLoS One. 2010;5:e9490.

9. Bokulich NA, Kaehler BD, Rideout JR, Dillon M, Bolyen E, Knight R, et al. Optimizing taxonomic classification of marker-gene amplicon sequences with QIIME 2’s q2-feature-classifier plugin. Microbiome. Microbiome; 2018;6:90.

10. DeSantis TZ, Hugenholtz P, Larsen N, Rojas M, Brodie EL, Keller K, et al. Greengenes, a chimera-checked 16S rRNA gene database and workbench compatible with ARB. Appl Environ Microbiol. 2006;72:5069–72.

11. McDonald D, Price MN, Goodrich J, Nawrocki EP, Desantis TZ, Probst A, et al. An improved Greengenes taxonomy with explicit ranks for ecological and evolutionary analyses of bacteria and archaea. ISME J [Internet]. Nature Publishing Group; 2012;6:610–8. Available from: http://dx.doi.org/10.1038/ismej.2011.139

12. Bokulich NA, Subramanian S, Faith JJ, Gevers D, Gordon JI, Knight R, et al. Quality-filtering vastly improves diversity estimates from Illumina amplicon sequencing. Nat Methods. 2013;10:57–9.

13. Zaneveld JR, McMinds R, Vega Thurber R. Stress and stability: applying the Anna Karenina principle to animal microbiomes. Nat Microbiol. 2017;2:17121.

14. Jin Song S, Woodhams DC, Martino C, Allaband C, Mu A, Javorschi-Miller-Montgomery S, et al. Engineering the microbiome for animal health and conservation. Exp Biol Med. 2019;244:494–504.

15. Kriss M, Hazleton KZ, Nusbacher NM, Martin CG, Lozupone CA. Low diversity gut microbiota dysbiosis: drivers, functional implications and recovery. Curr Opin Microbiol. Elsevier Ltd; 2018;44:34–40.

16. Woodhams DC, Alford RA, Antwis RE, Archer H, Becker MH, Belden LK, et al. Antifungal isolates database of amphibian skin-associated bacteria and function against emerging fungal pathogens. Ecology [Internet]. 2015;96:595–595. Available from: http://doi.wiley.com/10.1890/14-1837.1

17. Oksanen J, Blanchet F, Kindt R, Legendre P, O’hara R, Simpson G, et al. vegan: community ecology package. R package version 2.4-1. https://CRAN.R-project.org/package=vegan. [Internet]. 2016 [cited 2017 Sep 27]. Available from: https://scholar.google.com/scholar?hl=en&as_sdt=0%2C11&q=vegan%3A+community+ecology+package&btnG=

18. R Core Team. R: A language and environment for statistical computing. R Foundation for Statistical Computing. Vienna, Austria. URL https://www.R-project.org/. Vienna, Austria; 2018.

19. Revell LJ. phytools: an R package for phylogenetic comparative biology (and other things). Methods Ecol Evol. 2012;3:217–23.

20. Segata N, Izard J, Waldron L, Gevers D, Miropolsky L, Garrett WS, et al. Metagenomic biomarker discovery and explanation. Genome Biol. 2011;12:R60.

21. Afgan E, Baker D, Batut B, Van Den Beek M, Bouvier D, Ech M, et al. The Galaxy platform for accessible, reproducible and collaborative biomedical analyses: 2018 update. Nucleic Acids Res. 2018;46:W537–44.

22. JMP. Version 14.0.0. SAS Institute Inc. Cary, NC. 2019.

23. Ju F, Xia Y, Guo F, Wang Z, Zhang T. Taxonomic relatedness shapes bacterial assembly in activated sludge of globally distributed wastewater treatment plants. Environ Microbiol. 2014;16:2421–32.

24. Harrell Jr. FE, Dupont MC. The Hmisc package. 2006.

25. Benjamini Y, Hochberg Y. Controlling the false discovery rate : A practical and powerful approach to multiple testing. J R Stat Soc Ser B. 1995;57:289–300.

26. Csardi G, Nepusz T. The igraph software package for complex network research [Internet]. 2006. Available from: http://igraph.sf.net

27. Martín González AM, Dalsgaard B, Olesen JM. Centrality measures and the importance of generalist species in pollination networks. Ecol Complex. 2010;7:36–43.

28. Trosvik P, de Muinck EJ. Ecology of bacteria in the human gastrointestinal tract--identification of keystone and foundation taxa. Microbiome [Internet]. Microbiome; 2015;3:44. Available from: http://dx.doi.org/10.1186/s40168-015-0107-4

29. Erdos P, Renyi A. On the evolution of random graphs. Publ Math Inst Hung Acad Sci [Internet]. 1960;5:17–60. Available from: https://pdfs.semanticscholar.org/4201/73e087bca0bdb31985e28ff69c60a129c8ef.pdf


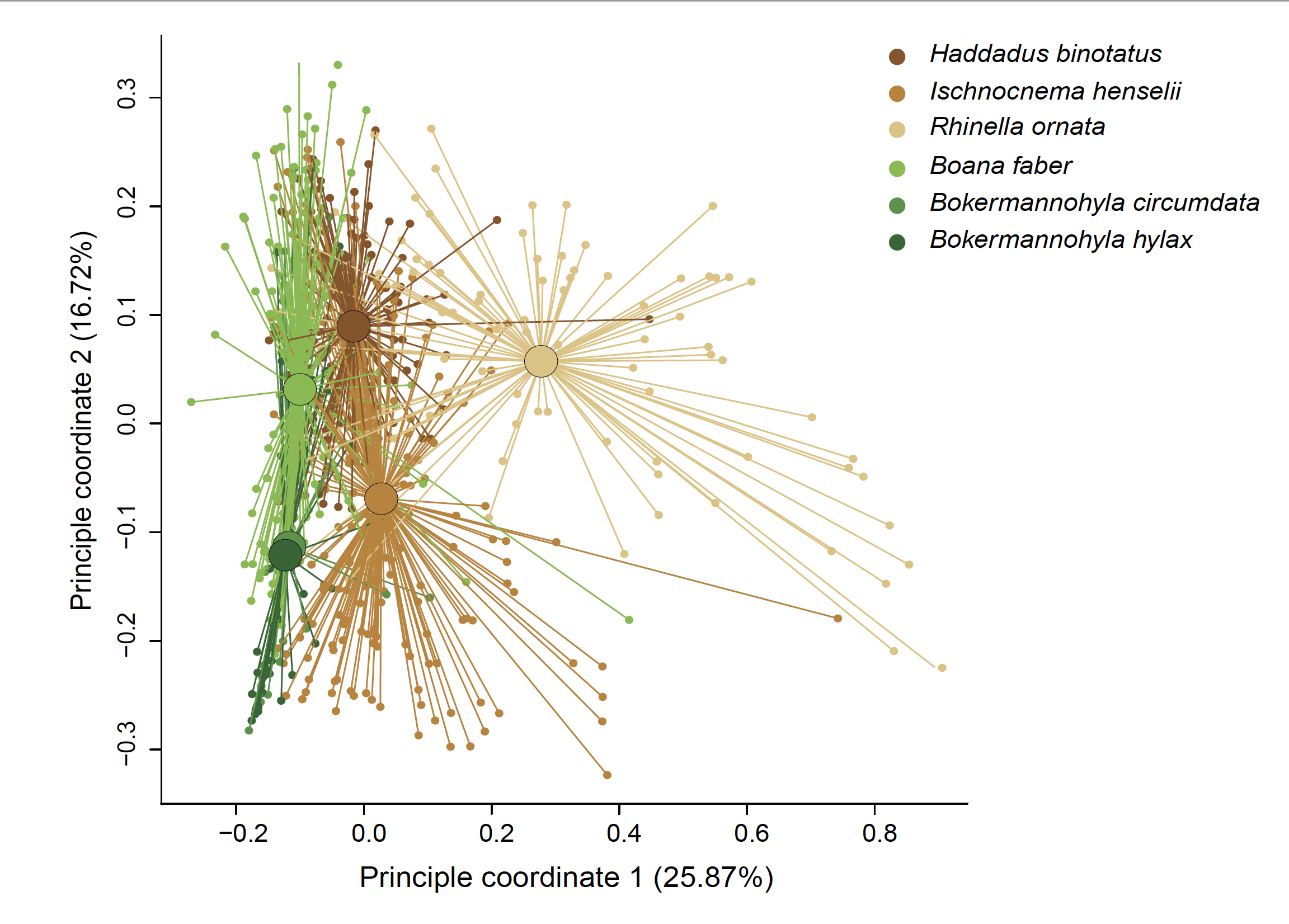


Supplementary Figure 1. Community composition of host skin bacteria calculated using principal coordinates analysis based on weighted UniFrac distances. Small circles indicate data points. Large circles indicate group centroids. Lines indicate distance to group centroids.


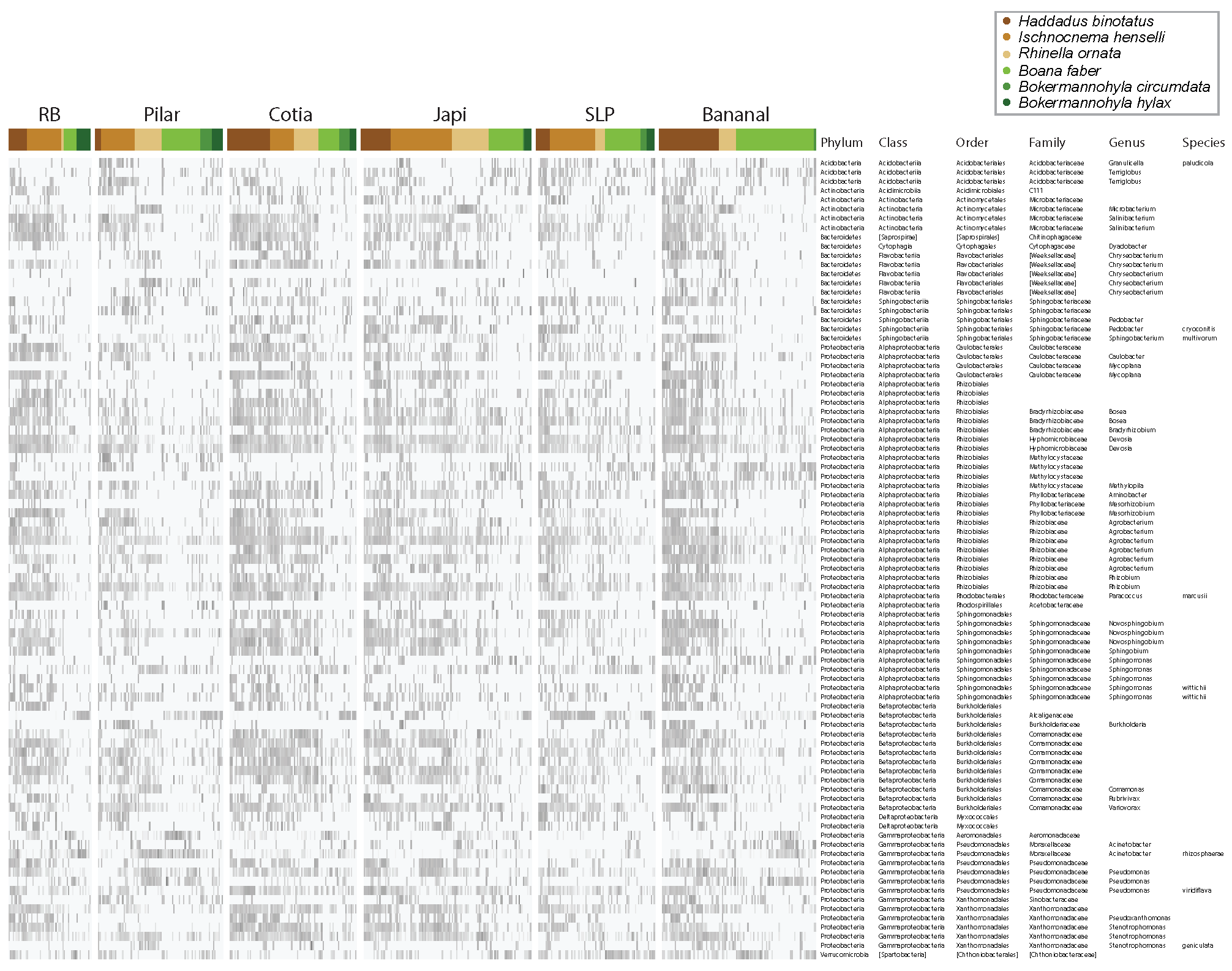


Supplementary Figure 2. Heatmap indicating bacterial taxa driving differences in skin bacterial community composition among host species at each of six study landscapes. Rows represent indicator sOTUs. Some sOTUs could not be identified to all taxonomic ranks. Columns represent individual samples. Darker shading indicates higher relative read abundance.


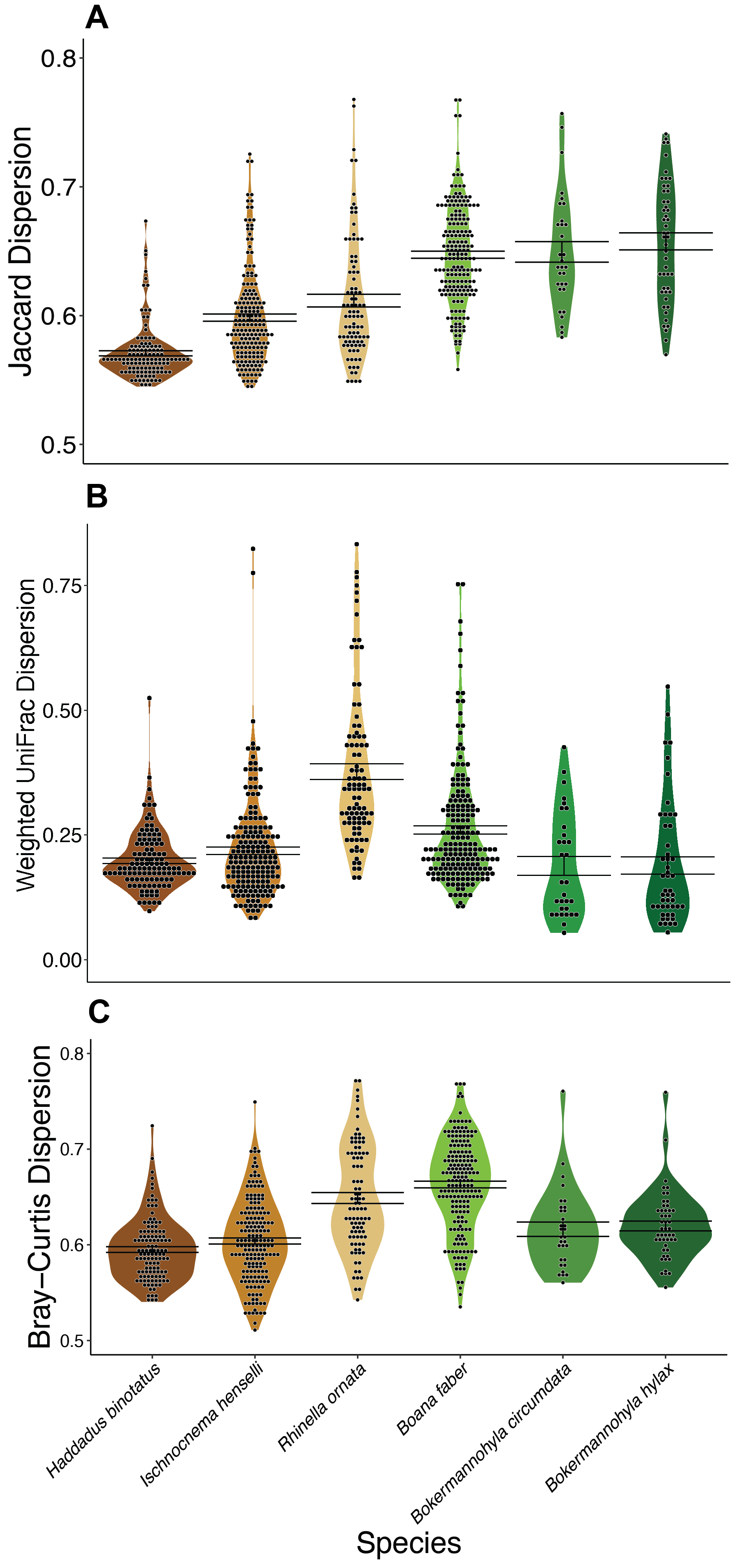


Supplementary Figure 3. For six anural species sampled in Brazil’s Atlantic Forest, violin plots showing likelihood of different levels of skin bacterial dispersion (distance to group centroid) based on A) Jaccard, B) weighted UniFrac, and C) Bray-Curtis distances. Bars on violin plots indicate mean ± standard error.


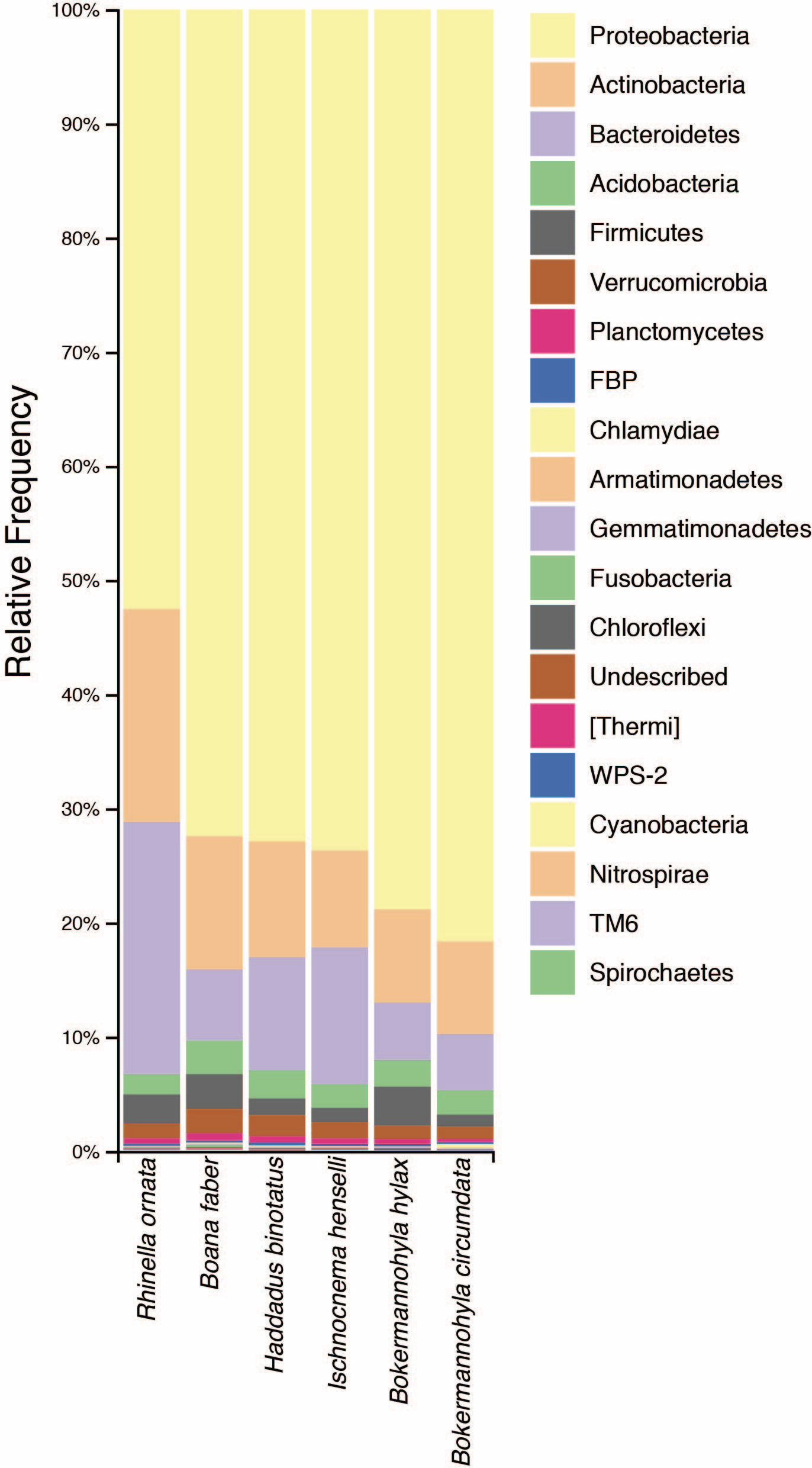


Supplementary Figure 4. Barplots indicating dominant bacterial phyla detected in the skin microbiome

of each host species.

Supplementary Table 1. Number and percentage of Bd-negative and Bd-positive individuals of each host species sampled at each study landscape. The full dataset is summarized at left. The subset of samples included in analyses following rarefaction is summarized at right. Totals for each species are bolded.

|  | All samples | | | | | | |  | Included after rarefaction | | | | | | |
| --- | --- | --- | --- | --- | --- | --- | --- | --- | --- | --- | --- | --- | --- | --- | --- |
|  | Bd-negative | |  | Bd-positive | |  | Grand Total |  | Bd-negative | |  | Bd-positive | |  | Grand Total |
|  | n | % |  | n | % |  |  |  | n | % |  | n | % |  |  |
| ***Boana faber*** | **307** | **86.2** |  | **49** | **13.8** |  | **356** |  | **158** | **82.7** |  | **33** | **17.3** |  | **191** |
| Bananal | 91 | 95.8 |  | 4 | 4.2 |  | 95 |  | 52 | 94.5 |  | 3 | 5.5 |  | 55 |
| Cotia | 40 | 88.9 |  | 5 | 11.1 |  | 45 |  | 13 | 86.7 |  | 2 | 13.3 |  | 15 |
| Intervales State Park | 15 | 71.4 |  | 6 | 28.6 |  | 21 |  | 8 | 80.0 |  | 2 | 20.0 |  | 10 |
| Serra do Japi | 35 | 79.5 |  | 9 | 20.5 |  | 44 |  | 17 | 70.8 |  | 7 | 29.2 |  | 24 |
| Pilar do Sul | 37 | 97.4 |  | 1 | 2.6 |  | 38 |  | 26 | 96.3 |  | 1 | 3.7 |  | 27 |
| Ribeirão Branco | 15 | 93.8 |  | 1 | 6.3 |  | 16 |  | 8 | 88.9 |  | 1 | 11.1 |  | 9 |
| São Luiz do Paraitinga | 54 | 100.0 |  | 0 | 0.0 |  | 54 |  | 25 | 100.0 |  | 0 | 0.0 |  | 25 |
| Núcleo Santa Virgínia | 20 | 46.5 |  | 23 | 53.5 |  | 43 |  | 9 | 34.6 |  | 17 | 65.4 |  | 26 |
| ***Bokermannohyla circumdata*** | **39** | **92.9** |  | **3** | **7.1** |  | **42** |  | **28** | **90.3** |  | **3** | **9.7** |  | **31** |
| Bananal | 1 | 100.0 |  | 0 | 0.0 |  | 1 |  | 1 | 100.0 |  | 0 | 0.0 |  | 1 |
| Cotia | 10 | 100.0 |  | 0 | 0.0 |  | 10 |  | 7 | 100.0 |  | 0 | 0.0 |  | 7 |
| Intervales State Park | 4 | 100.0 |  | 0 | 0.0 |  | 4 |  | 3 | 100.0 |  | 0 | 0.0 |  | 3 |
| Serra do Japi | 3 | 100.0 |  | 0 | 0.0 |  | 3 |  | 1 | 100.0 |  | 0 | 0.0 |  | 1 |
| Pilar do Sul | 7 | 77.8 |  | 2 | 22.2 |  | 9 |  | 6 | 75.0 |  | 2 | 25.0 |  | 8 |
| São Luiz do Paraitinga | 4 | 100.0 |  | 0 | 0.0 |  | 4 |  | 4 | 100.0 |  | 0 | 0.0 |  | 4 |
| Núcleo Santa Virgínia | 10 | 90.9 |  | 1 | 9.1 |  | 11 |  | 6 | 85.7 |  | 1 | 14.3 |  | 7 |
| ***Bokermannohyla hylax*** | **55** | **87.3** |  | **8** | **12.7** |  | **63** |  | **42** | **85.7** |  | **7** | **14.3** |  | **49** |
| Cotia | 5 | 83.3 |  | 1 | 16.7 |  | 6 |  | 5 | 100.0 |  | 0 | 0.0 |  | 5 |
| Serra do Japi | 7 | 100.0 |  | 0 | 0.0 |  | 7 |  | 5 | 100.0 |  | 0 | 0.0 |  | 5 |
| Pilar do Sul | 9 | 100.0 |  | 0 | 0.0 |  | 9 |  | 8 | 100.0 |  | 0 | 0.0 |  | 8 |
| Ribeirão Branco | 11 | 100.0 |  | 0 | 0.0 |  | 11 |  | 10 | 100.0 |  | 0 | 0.0 |  | 10 |
| São Luiz do Paraitinga | 8 | 100.0 |  | 0 | 0.0 |  | 8 |  | 6 | 100.0 |  | 0 | 0.0 |  | 6 |
| Núcleo Santa Virgínia | 15 | 68.2 |  | 7 | 31.8 |  | 22 |  | 8 | 53.3 |  | 7 | 46.7 |  | 15 |
| ***Haddadus binotatus*** | **126** | **100.0** |  | **0** | **0.0** |  | **126** |  | **120** | **100.0** |  | **0** | **0.0** |  | **120** |
| Bananal | 46 | 100.0 |  | 0 | 0.0 |  | 46 |  | 42 | 100.0 |  | 0 | 0.0 |  | 42 |
| Cotia | 31 | 100.0 |  | 0 | 0.0 |  | 31 |  | 30 | 100.0 |  | 0 | 0.0 |  | 30 |
| Serra do Japi | 21 | 100.0 |  | 0 | 0.0 |  | 21 |  | 21 | 100.0 |  | 0 | 0.0 |  | 21 |
| Pilar do Sul | 4 | 100.0 |  | 0 | 0.0 |  | 4 |  | 4 | 100.0 |  | 0 | 0.0 |  | 4 |
| Ribeirão Branco | 14 | 100.0 |  | 0 | 0.0 |  | 14 |  | 13 | 100.0 |  | 0 | 0.0 |  | 13 |
| São Luiz do Paraitinga | 10 | 100.0 |  | 0 | 0.0 |  | 10 |  | 10 | 100.0 |  | 0 | 0.0 |  | 10 |
| ***Ischnocnema henselii*** | **133** | **65.2** |  | **71** | **34.8** |  | **204** |  | **116** | **63.7** |  | **66** | **36.3** |  | **182** |
| Cotia | 15 | 88.2 |  | 2 | 11.8 |  | 17 |  | 15 | 88.2 |  | 2 | 11.8 |  | 17 |
| Intervales State Park | 17 | 63.0 |  | 10 | 37.0 |  | 27 |  | 15 | 62.5 |  | 9 | 37.5 |  | 24 |
| Serra do Japi | 27 | 61.4 |  | 17 | 38.6 |  | 44 |  | 27 | 62.8 |  | 16 | 37.2 |  | 43 |
| Pilar do Sul | 20 | 60.6 |  | 13 | 39.4 |  | 33 |  | 12 | 50.0 |  | 12 | 50.0 |  | 24 |
| Ribeirão Branco | 20 | 74.1 |  | 7 | 25.9 |  | 27 |  | 18 | 75.0 |  | 6 | 25.0 |  | 24 |
| São Luiz do Paraitinga | 21 | 60.0 |  | 14 | 40.0 |  | 35 |  | 18 | 56.3 |  | 14 | 43.8 |  | 32 |
| Núcleo Santa Virgínia | 13 | 61.9 |  | 8 | 38.1 |  | 21 |  | 11 | 61.1 |  | 7 | 38.9 |  | 18 |
| ***Rhinella ornata*** | **106** | **94.6** |  | **6** | **5.4** |  | **112** |  | **90** | **96.8** |  | **3** | **3.2** |  | **93** |
| Bananal | 13 | 92.9 |  | 1 | 7.1 |  | 14 |  | 11 | 91.7 |  | 1 | 8.3 |  | 12 |
| Cotia | 17 | 85.0 |  | 3 | 15.0 |  | 20 |  | 16 | 94.1 |  | 1 | 5.9 |  | 17 |
| Intervales State Park | 14 | 100.0 |  | 0 | 0.0 |  | 14 |  | 10 | 100.0 |  | 0 | 0.0 |  | 10 |
| Serra do Japi | 30 | 96.8 |  | 1 | 3.2 |  | 31 |  | 25 | 96.2 |  | 1 | 3.8 |  | 26 |
| Pilar do Sul | 22 | 100.0 |  | 0 | 0.0 |  | 22 |  | 19 | 100.0 |  | 0 | 0.0 |  | 19 |
| Ribeirão Branco | 2 | 66.7 |  | 1 | 33.3 |  | 3 |  | 2 | 100.0 |  | 0 | 0.0 |  | 2 |
| São Luiz do Paraitinga | 8 | 100.0 |  | 0 | 0.0 |  | 8 |  | 7 | 100.0 |  | 0 | 0.0 |  | 7 |
| **Grand Total** | **766** | **84.8** |  | **137** | **15.2** |  | **903** |  | **554** | **83.2** |  | **112** | **16.8** |  | **666** |

Supplementary Table 2. Results of Tukey’s HSD pairwise comparison on alpha diversity metrics between species. Significant *p*-values are bolded.

|  | | Observed sOTUs | | | | Faith’s phylogenetic diversity | | | | Shannon diversity index | | | |
| --- | --- | --- | --- | --- | --- | --- | --- | --- | --- | --- | --- | --- | --- |
| Comparison | | Difference | SE | t | *p* | Difference | SE | t | p | Difference | SE | t | *p* |
| *Bo. faber* | *Bk. circumdata* | 18.163 | 14.50 | 1.25 | 0.8105 | 0.93436 | 1.00 | 0.93 | 0.9380 | 1.35331 | 0.32 | 4.22 | **0.0004** |
| *Bo. faber* | *Bk. hylax* | 21.307 | 12.83 | 1.66 | 0.5582 | 0.79118 | 0.90 | 0.88 | 0.9507 | 1.42194 | 0.29 | 4.95 | **<.0001** |
| *Bo. faber* | *H. binotatus* | -121.816 | 9.16 | -13.29 | **<.0001** | -7.71626 | 0.64 | -12.11 | **<.0001** | -1.83480 | 0.20 | -9.01 | **<.0001** |
| *Bo. faber* | *I. henselii* | -61.187 | 8.49 | -7.20 | **<.0001** | -4.17039 | 0.59 | -7.07 | **<.0001** | 0.13160 | 0.19 | 0.70 | 0.9823 |
| *Bo. faber* | *R. ornata* | -28.869 | 9.86 | -2.93 | **0.0417** | -0.93166 | 0.68 | -1.36 | 0.7482 | -0.07189 | 0.22 | -0.33 | 0.9995 |
| *Bk. circumdata* | *Bk. hylax* | 3.144 | 17.62 | 0.18 | 1.0000 | -0.14318 | 1.23 | -0.12 | 1.0000 | 0.06863 | 0.39 | 0.17 | 1.0000 |
| *Bk. circumdata* | *H. binotatus* | -139.979 | 15.56 | -9.00 | **<.0001** | -8.65062 | 1.08 | -8.02 | **<.0001** | -3.18810 | 0.34 | -9.25 | **<.0001** |
| *Bk. circumdata* | *I. henselii* | -79.351 | 14.72 | -5.39 | **<.0001** | -5.10475 | 1.02 | -5.01 | **<.0001** | -1.22171 | 0.33 | -3.75 | **0.0027** |
| *Bk. circumdata* | *R. ornata* | -47.032 | 15.69 | -3.00 | **0.0339** | -1.86602 | 1.08 | -1.72 | 0.5189 | -1.42519 | 0.35 | -4.11 | **0.0007** |
| *Bk. hylax* | *H. binotatus* | -143.123 | 13.81 | -10.36 | **<.0001** | -8.50744 | 0.97 | -8.80 | **<.0001** | -3.25674 | 0.31 | -10.55 | **<.0001** |
| *Bk. hylax* | *I. henselii* | -82.494 | 12.84 | -6.42 | **<.0001** | -4.96157 | 0.90 | -5.52 | **<.0001** | -1.29035 | 0.29 | -4.49 | **0.0001** |
| *Bk. hylax* | *R. ornata* | -50.176 | 14.20 | -3.53 | **0.0060** | -1.72284 | 0.99 | -1.73 | 0.5102 | -1.49383 | 0.32 | -4.71 | **<.0001** |
| *H. binotatus* | *I. henselii* | 60.629 | 9.65 | 6.28 | **<.0001** | 3.54587 | 0.67 | 5.28 | **<.0001** | 1.96639 | 0.21 | 9.17 | **<.0001** |
| *H. binotatus* | *R. ornata* | 92.947 | 10.69 | 8.69 | **<.0001** | 6.78460 | 0.74 | 9.16 | **<.0001** | 1.76291 | 0.24 | 7.44 | **<.0001** |
| *I. henselii* | *R. ornata* | 32.318 | 9.82 | 3.29 | **0.0136** | 3.23873 | 0.68 | 4.76 | **<.0001** | -0.20348 | 0.22 | -0.94 | 0.9373 |

Supplementary Table 3. Results of pairwise PermANOVA comparison on beta diversity metrics between species. Significant *p*-values are bolded.

|  |  | Unweighted UniFrac | | | | Weighted UniFrac | | | |
| --- | --- | --- | --- | --- | --- | --- | --- | --- | --- |
| Comparison | | F | R^2^ | *p* | *p*-adj | F | R^2^ | *p* | *p*-adj |
| *Bo. faber* | *I. henselii* | 114.88 | 0.24 | 0.001 | **0.002** | 59.06 | 0.14 | 0.001 | **0.001** |
| *Bo. faber* | *R. ornata* | 19.59 | 0.06 | 0.001 | **0.002** | 102.80 | 0.27 | 0.001 | **0.001** |
| *Bo. faber* | *H .binotatus* | 160.06 | 0.34 | 0.001 | **0.002** | 50.06 | 0.14 | 0.001 | **0.001** |
| *Bo. faber* | *Bk. hylax* | 2.10 | 0.01 | 0.134 | 0.155 | 17.80 | 0.07 | 0.001 | **0.001** |
| *Bo. faber* | *Bk. circumdata* | 0.68 | 0.00 | 0.470 | 0.504 | 11.96 | 0.05 | 0.001 | **0.001** |
| *I. henselii* | *R. ornata* | 32.15 | 0.11 | 0.001 | **0.002** | 124.31 | 0.31 | 0.001 | **0.001** |
| *I. henselii* | *H. binotatus* | 14.07 | 0.04 | 0.001 | **0.002** | 48.20 | 0.14 | 0.001 | **0.001** |
| *I. henselii* | *Bk. hylax* | 46.93 | 0.17 | 0.001 | **0.002** | 20.65 | 0.08 | 0.001 | **0.001** |
| *I. henselii* | *Bk. circumdata* | 34.21 | 0.14 | 0.001 | **0.002** | 13.69 | 0.06 | 0.001 | **0.001** |
| *R. ornata* | *H. binotatus* | 62.41 | 0.23 | 0.001 | **0.002** | 134.29 | 0.39 | 0.001 | **0.001** |
| *R. ornata* | *Bk. hylax* | 10.44 | 0.07 | 0.002 | **0.003** | 61.07 | 0.30 | 0.001 | **0.001** |
| *R. ornata* | *Bk. circumdata* | 7.45 | 0.06 | 0.003 | **0.004** | 43.99 | 0.27 | 0.001 | **0.001** |
| *H. binotatus* | *Bk. hylax* | 81.79 | 0.33 | 0.001 | **0.002** | 74.75 | 0.31 | 0.001 | **0.001** |
| *H. binotatus* | *Bk. circumdata* | 66.64 | 0.31 | 0.001 | **0.002** | 53.39 | 0.26 | 0.001 | **0.001** |
| *Bk. hylax* | *Bk. circumdata* | 0.34 | 0.00 | 0.653 | 0.653 | 0.33 | 0.00 | 0.778 | 0.778 |

Supplementary Table 4. Differentially abundant skin bacterial sOTUs for six amphibian species identified at each of six study landscapes with LEfSe analysis.

| **Landscape: Bananal** | | |  |  |  |  |
| --- | --- | --- | --- | --- | --- | --- |
| Host | Phylum | Class | Order | Family | Genus | Species |
| *Bk. circumdata* | Proteobacteria | Gammaproteobacteria | Pseudomonadales | Moraxellaceae | *Acinetobacter* |  |
| *H. binotatus* | Actinobacteria | Actinobacteria | Actinomycetales | Microbacteriaceae | *Salinibacterium* |  |
| *H. binotatus* | Actinobacteria | Actinobacteria | Actinomycetales | Microbacteriaceae |  |  |
| *H. binotatus* | Bacteroidetes | [Saprospirae] | [Saprospirales] | Chitinophagaceae |  |  |
| *H. binotatus* | Bacteroidetes | Cytophagia | Cytophagales | Cytophagaceae | *Dyadobacter* |  |
| *H. binotatus* | Bacteroidetes | Flavobacteriia | Flavobacteriales | [Weeksellaceae] | *Chryseobacterium* |  |
| *H. binotatus* | Bacteroidetes | Flavobacteriia | Flavobacteriales | [Weeksellaceae] | *Chryseobacterium* |  |
| *H. binotatus* | Bacteroidetes | Sphingobacteriia | Sphingobacteriales | Sphingobacteriaceae |  |  |
| *H. binotatus* | Bacteroidetes | Sphingobacteriia | Sphingobacteriales | Sphingobacteriaceae | *Pedobacter* |  |
| *H. binotatus* | Bacteroidetes | Sphingobacteriia | Sphingobacteriales | Sphingobacteriaceae | *Sphingobacterium* | *multivorum* |
| *H. binotatus* | Proteobacteria | Alphaproteobacteria | Caulobacterales | Caulobacteraceae | *Mycoplana* |  |
| *H. binotatus* | Proteobacteria | Alphaproteobacteria | Caulobacterales | Caulobacteraceae | *Mycoplana* |  |
| *H. binotatus* | Proteobacteria | Alphaproteobacteria | Rhizobiales |  |  |  |
| *H. binotatus* | Proteobacteria | Alphaproteobacteria | Rhizobiales | Hyphomicrobiaceae | *Devosia* |  |
| *H. binotatus* | Proteobacteria | Alphaproteobacteria | Rhizobiales | Methylocystaceae | *Methylopila* |  |
| *H. binotatus* | Proteobacteria | Alphaproteobacteria | Rhizobiales | Phyllobacteriaceae | *Aminobacter* |  |
| *H. binotatus* | Proteobacteria | Alphaproteobacteria | Rhizobiales | Phyllobacteriaceae | *Mesorhizobium* |  |
| *H. binotatus* | Proteobacteria | Alphaproteobacteria | Rhizobiales | Rhizobiaceae | *Agrobacterium* |  |
| *H. binotatus* | Proteobacteria | Alphaproteobacteria | Rhizobiales | Rhizobiaceae | *Agrobacterium* |  |
| *H. binotatus* | Proteobacteria | Alphaproteobacteria | Rhizobiales | Rhizobiaceae | *Agrobacterium* |  |
| *H. binotatus* | Proteobacteria | Alphaproteobacteria | Rhizobiales | Rhizobiaceae | *Agrobacterium* |  |
| *H. binotatus* | Proteobacteria | Alphaproteobacteria | Rhizobiales | Rhizobiaceae | *Rhizobium* |  |
| *H. binotatus* | Proteobacteria | Alphaproteobacteria | Rhizobiales |  |  |  |
| *H. binotatus* | Proteobacteria | Alphaproteobacteria | Rhodobacterales | Rhodobacteraceae | *Paracoccus* | *marcusii* |
| *H. binotatus* | Proteobacteria | Alphaproteobacteria | Sphingomonadales | Sphingomonadaceae | *Novosphingobium* |  |
| *H. binotatus* | Proteobacteria | Alphaproteobacteria | Sphingomonadales | Sphingomonadaceae | *Novosphingobium* |  |
| *H. binotatus* | Proteobacteria | Alphaproteobacteria | Sphingomonadales | Sphingomonadaceae | *Novosphingobium* |  |
| *H. binotatus* | Proteobacteria | Alphaproteobacteria | Sphingomonadales | Sphingomonadaceae | *Sphingobium* |  |
| *H. binotatus* | Proteobacteria | Alphaproteobacteria | Sphingomonadales | Sphingomonadaceae | *Sphingomonas* |  |
| *H. binotatus* | Proteobacteria | Alphaproteobacteria | Sphingomonadales | Sphingomonadaceae | *Sphingomonas* | *wittichii* |
| *H. binotatus* | Proteobacteria | Betaproteobacteria | Burkholderiales |  |  |  |
| *H. binotatus* | Proteobacteria | Betaproteobacteria | Burkholderiales | Comamonadaceae | *Comamonas* |  |
| *H. binotatus* | Proteobacteria | Betaproteobacteria | Burkholderiales | Comamonadaceae | *Variovorax* |  |
| *H. binotatus* | Proteobacteria | Betaproteobacteria | Burkholderiales | Comamonadaceae |  |  |
| *H. binotatus* | Proteobacteria | Betaproteobacteria | Burkholderiales | Comamonadaceae |  |  |
| *H. binotatus* | Proteobacteria | Betaproteobacteria | Burkholderiales | Comamonadaceae |  |  |
| *H. binotatus* | Proteobacteria | Betaproteobacteria | Burkholderiales | Comamonadaceae |  |  |
| *H. binotatus* | Proteobacteria | Gammaproteobacteria | Pseudomonadales | Pseudomonadaceae |  |  |
| *H. binotatus* | Proteobacteria | Gammaproteobacteria | Pseudomonadales | Pseudomonadaceae | *Pseudomonas* | *viridiflava* |
| *H. binotatus* | Proteobacteria | Gammaproteobacteria | Xanthomonadales | Xanthomonadaceae |  |  |
| *R. ornata* | Actinobacteria | Actinobacteria | Actinomycetales | Microbacteriaceae | *Microbacterium* |  |
| *R. ornata* | Bacteroidetes | Flavobacteriia | Flavobacteriales | [Weeksellaceae] | *Chryseobacterium* |  |
| *R. ornata* | Proteobacteria | Gammaproteobacteria | Xanthomonadales | Xanthomonadaceae | *Stenotrophomonas* | *geniculata* |
|  |  |  |  |  |  |  |
| **Landscape: Cotia** | | |  |  |  |  |
| Host | Phylum | Class | Order | Family | Genus | Species |
| *R. ornata* | Proteobacteria | Betaproteobacteria | Burkholderiales | Comamonadaceae |  |  |
|  |  |  |  |  |  |  |
| **Landscape: Serra do Japi** | |  |  |  |  |  |
| Host | Phylum | Class | Order | Family | Genus | Species |
| *H. binotatus* | Bacteroidetes | Flavobacteriia | Flavobacteriales | [Weeksellaceae] | *Chryseobacterium* |  |
| *H. binotatus* | Proteobacteria | Alphaproteobacteria | Sphingomonadales | Sphingomonadaceae | *Novosphingobium* |  |
|  |  |  |  |  |  |  |
| **Landscape: Pilar do Sul** | |  |  |  |  |  |
| Host | Phylum | Class | Order | Family | Genus | Species |
| *Bo. faber* | Proteobacteria | Alphaproteobacteria | Sphingomonadales | Sphingomonadaceae | *Sphingomonas* |  |
| *Bo. faber* | Proteobacteria | Gammaproteobacteria | Aeromonadales | Aeromonadaceae |  |  |
| *Bo. faber* | Proteobacteria | Gammaproteobacteria | Pseudomonadales | Pseudomonadaceae | *Pseudomonas* |  |
| *Bk. circumdata* | Proteobacteria | Betaproteobacteria | Burkholderiales | Comamonadaceae | *Rubrivivax* |  |
| *H. binotatus* | Actinobacteria | Actinobacteria | Actinomycetales | Microbacteriaceae | *Salinibacterium* |  |
| *H. binotatus* | Proteobacteria | Alphaproteobacteria | Rhizobiales | Hyphomicrobiaceae | *Devosia* |  |
| *H. binotatus* | Proteobacteria | Alphaproteobacteria | Rhizobiales | Methylocystaceae |  |  |
| *H. binotatus* | Proteobacteria | Alphaproteobacteria | Rhizobiales | Phyllobacteriaceae | *Aminobacter* |  |
| *H. binotatus* | Proteobacteria | Alphaproteobacteria | Rhizobiales | Rhizobiaceae | *Rhizobium* |  |
| *H. binotatus* | Proteobacteria | Betaproteobacteria | Burkholderiales | Comamonadaceae | *Variovorax* |  |
| *H. binotatus* | Proteobacteria | Gammaproteobacteria | Xanthomonadales | Sinobacteraceae |  |  |
| *H. binotatus* | Proteobacteria | Gammaproteobacteria | Xanthomonadales | Xanthomonadaceae | *Stenotrophomonas* |  |
| *I. henselii* | Bacteroidetes | Sphingobacteriia | Sphingobacteriales | Sphingobacteriaceae |  |  |
| *I. henselii* | Proteobacteria | Alphaproteobacteria | Rhizobiales | Bradyrhizobiaceae | *Bosea* |  |
| *I. henselii* | Proteobacteria | Alphaproteobacteria | Rhizobiales | Methylocystaceae |  |  |
| *I. henselii* | Proteobacteria | Alphaproteobacteria | Rhizobiales | Rhizobiaceae | *Agrobacterium* |  |
| *I. henselii* | Proteobacteria | Betaproteobacteria | Burkholderiales | Burkholderiaceae | *Burkholderia* |  |
| *R. ornata* | Bacteroidetes | Flavobacteriia | Flavobacteriales | [Weeksellaceae] | *Chryseobacterium* |  |
| *R. ornata* | Bacteroidetes | Flavobacteriia | Flavobacteriales | [Weeksellaceae] | *Chryseobacterium* |  |
| *R. ornata* | Proteobacteria | Alphaproteobacteria | Rhizobiales | Rhizobiaceae | *Agrobacterium* |  |
| *R. ornata* | Proteobacteria | Gammaproteobacteria | Pseudomonadales | Pseudomonadaceae | *Pseudomonas* |  |
|  |  |  |  |  |  |  |
| **Landscape: Ribeirão Branco** | |  |  |  |  |  |
| Host | Phylum | Class | Order | Family | Genus | Species |
| *Bk. hylax* | Proteobacteria | Betaproteobacteria | Burkholderiales | Alcaligenaceae |  |  |
| *Bk. hylax* | Proteobacteria | Gammaproteobacteria | Pseudomonadales | Moraxellaceae | *Acinetobacter* | *rhizosphaerae* |
| *H. binotatus* | Proteobacteria | Alphaproteobacteria | Caulobacterales | Caulobacteraceae | *Caulobacter* |  |
| *H. binotatus* | Actinobacteria | Actinobacteria | Actinomycetales | Microbacteriaceae | *Salinibacterium* |  |
| *H. binotatus* | Proteobacteria | Alphaproteobacteria | Rhizobiales | Bradyrhizobiaceae | *Bosea* |  |
| *H. binotatus* | Proteobacteria | Alphaproteobacteria | Rhizobiales | Bradyrhizobiaceae | *Bosea* |  |
| *H. binotatus* | Proteobacteria | Alphaproteobacteria | Rhizobiales | Hyphomicrobiaceae | *Devosia* |  |
| *H. binotatus* | Proteobacteria | Alphaproteobacteria | Rhizobiales | Rhizobiaceae | *Agrobacterium* |  |
| *H. binotatus* | Proteobacteria | Alphaproteobacteria | Rhizobiales | Rhizobiaceae | *Agrobacterium* |  |
| *H. binotatus* | Proteobacteria | Alphaproteobacteria | Rhizobiales | Rhizobiaceae | *Rhizobium* |  |
| *H. binotatus* | Proteobacteria | Alphaproteobacteria | Sphingomonadales | Sphingomonadaceae | *Novosphingobium* |  |
| *H. binotatus* | Proteobacteria | Betaproteobacteria | Burkholderiales | Comamonadaceae |  |  |
| *H. binotatus* | Proteobacteria | Gammaproteobacteria | Xanthomonadales | Xanthomonadaceae | *Pseudoxanthomonas* |  |
| *H. binotatus* | Proteobacteria | Gammaproteobacteria | Xanthomonadales | Xanthomonadaceae | *Stenotrophomonas* |  |
|  |  |  |  |  |  |  |
| **Landscape: São Luís do Paraitinga** | | |  |  |  |  |
| Host | Phylum | Class | Order | Family | Genus | Species |
| *Bo. faber* | Proteobacteria | Alphaproteobacteria | Rhizobiales | Bradyrhizobiaceae | *Bradyrhizobium* |  |
| *Bk. circumdata* | Proteobacteria | Betaproteobacteria | Burkholderiales | Alcaligenaceae |  |  |
| *H. binotatus* | Acidobacteria | Acidobacteriia | Acidobacteriales | Acidobacteriaceae | *Terriglobus* |  |
| *H. binotatus* | Actinobacteria | Acidimicrobiia | Acidimicrobiales | C111 |  |  |
| *H. binotatus* | Bacteroidetes | [Saprospirae] | [Saprospirales] | Chitinophagaceae |  |  |
| *H. binotatus* | Bacteroidetes | Sphingobacteriia | Sphingobacteriales | Sphingobacteriaceae | *Pedobacter* | *cryoconitis* |
| *H. binotatus* | Bacteroidetes | Sphingobacteriia | Sphingobacteriales | Sphingobacteriaceae | *Sphingobacterium* | *multivorum* |
| *H. binotatus* | Proteobacteria | Alphaproteobacteria | Caulobacterales | Caulobacteraceae |  |  |
| *H. binotatus* | Proteobacteria | Alphaproteobacteria | Rhizobiales |  |  |  |
| *H. binotatus* | Proteobacteria | Alphaproteobacteria | Rhizobiales | Bradyrhizobiaceae | *Bosea* |  |
| *H. binotatus* | Proteobacteria | Alphaproteobacteria | Rhizobiales | Hyphomicrobiaceae | *Devosia* |  |
| *H. binotatus* | Proteobacteria | Alphaproteobacteria | Rhizobiales | Hyphomicrobiaceae | *Devosia* |  |
| *H. binotatus* | Proteobacteria | Alphaproteobacteria | Rhizobiales | Methylocystaceae |  |  |
| *H. binotatus* | Proteobacteria | Alphaproteobacteria | Rhizobiales | Phyllobacteriaceae | *Aminobacter* |  |
| *H. binotatus* | Proteobacteria | Alphaproteobacteria | Rhizobiales | Phyllobacteriaceae | *Mesorhizobium* |  |
| *H. binotatus* | Proteobacteria | Alphaproteobacteria | Rhizobiales | Rhizobiaceae | *Agrobacterium* |  |
| *H. binotatus* | Proteobacteria | Alphaproteobacteria | Rhizobiales | Rhizobiaceae | *Agrobacterium* |  |
| *H. binotatus* | Proteobacteria | Alphaproteobacteria | Sphingomonadales |  |  |  |
| *H. binotatus* | Proteobacteria | Alphaproteobacteria | Sphingomonadales | Sphingomonadaceae | *Sphingomonas* | *wittichii* |
| *H. binotatus* | Proteobacteria | Betaproteobacteria | Burkholderiales | Comamonadaceae |  |  |
| *H. binotatus* | Proteobacteria | Betaproteobacteria | Burkholderiales | Comamonadaceae | *Variovorax* |  |
| *H. binotatus* | Proteobacteria | Deltaproteobacteria | Myxococcales |  |  |  |
| *H. binotatus* | Proteobacteria | Deltaproteobacteria | Myxococcales |  |  |  |

Supplementary Table 5. Results of Tukey’s HSD pairwise comparison of unweighted and weighted UniFrac dispersion between species. Significant *p*-values are bolded.

|  |  | Unweighted UniFrac Dispersion | | | | Weighted UniFrac Dispersion | | | |
| --- | --- | --- | --- | --- | --- | --- | --- | --- | --- |
| Comparison | | Difference | SE | t | *p* | Difference | SE | t | *p* |
| *Bo. faber* | *Bk. circumdata* | -0.000277 | 0.011872 | -0.02 | 1 | 0.070652 | 0.021584 | 3.27 | **0.0142** |
| *Bo. faber* | *Bk. hylax* | -0.010711 | 0.009742 | -1.1 | 0.8807 | 0.071599 | 0.018078 | 3.96 | **0.0012** |
| *Bo. faber* | *H. binotatus* | 0.079694 | 0.007043 | 11.31 | **<.0001** | 0.059136 | 0.013158 | 4.49 | **0.0001** |
| *Bo. faber* | *I. henselii* | 0.043261 | 0.006676 | 6.48 | **<.0001** | 0.039967 | 0.011931 | 3.35 | **0.0111** |
| *Bo. faber* | *R. ornata* | 0.022043 | 0.007948 | 2.77 | 0.0688 | -0.12269 | 0.014249 | -8.61 | **<.0001** |
| *Bk. circumdata* | *Bk. hylax* | -0.010434 | 0.013878 | -0.75 | 0.9748 | 0.000947 | 0.025433 | 0.04 | 1 |
| *Bk. circumdata* | *H. binotatus* | 0.079971 | 0.012318 | 6.49 | **<.0001** | -0.01152 | 0.0228 | -0.51 | 0.996 |
| *Bk. circumdata* | *I. henselii* | 0.043538 | 0.012055 | 3.61 | **0.0058** | -0.03069 | 0.021641 | -1.42 | 0.7161 |
| *Bk. circumdata* | *R. ornata* | 0.022319 | 0.012836 | 1.74 | 0.5091 | -0.19334 | 0.023105 | -8.37 | **<.0001** |
| *Bk. hylax* | *H. binotatus* | 0.090405 | 0.010251 | 8.82 | **<.0001** | -0.01246 | 0.019521 | -0.64 | 0.9881 |
| *Bk. hylax* | *I. henselii* | 0.053972 | 0.009783 | 5.52 | **<.0001** | -0.03163 | 0.018003 | -1.76 | 0.4945 |
| *Bk. hylax* | *R. ornata* | 0.032753 | 0.010677 | 3.07 | **0.0311** | -0.19428 | 0.020055 | -9.69 | **<.0001** |
| *H. binotatus* | *I. henselii* | -0.036433 | 0.007355 | -4.95 | **<.0001** | -0.01917 | 0.013815 | -1.39 | 0.7346 |
| *H. binotatus* | *R. ornata* | -0.057652 | 0.008553 | -6.74 | **<.0001** | -0.18182 | 0.01561 | -11.65 | **<.0001** |
| *I. henselii* | *R. ornata* | -0.021218 | 0.007684 | -2.76 | 0.0709 | -0.16265 | 0.014289 | -11.38 | **<.0001** |

Supplementary Table 6. Results of Tukey’s HSD pairwise comparison of Jaccard and Bray-Curtis dispersion between species. Significant *p*-values are bolded.

|  |  | Jaccard dispersion | | | | Bray-Curtis dispersion | | | |
| --- | --- | --- | --- | --- | --- | --- | --- | --- | --- |
| Comparison | | Difference | SE | t | *p* | Difference | SE | t | *p* |
| *Bo. faber* | *Bk. circumdata* | 0.006 | 0.009 | 0.65 | 0.987 | 0.183 | 0.021 | 8.91 | **<.0001** |
| *Bo. faber* | *Bk. hylax* | -0.005 | 0.007 | -0.77 | 0.973 | 0.209 | 0.017 | 12.1 | **<.0001** |
| *Bo. faber* | *H. binotatus* | 0.080 | 0.005 | 15.48 | **<.0001** | 0.089 | 0.013 | 7.11 | **<.0001** |
| *Bo. faber* | *I. henselii* | 0.038 | 0.005 | 7.99 | **<.0001** | 0.068 | 0.011 | 5.95 | **<.0001** |
| *Bo. faber* | *R. ornata* | 0.034 | 0.006 | 6.13 | **<.0001** | 0.053 | 0.014 | 3.89 | **0.002** |
| *Bk. circumdata* | *Bk. hylax* | -0.011 | 0.010 | -1.1 | 0.883 | 0.026 | 0.024 | 1.07 | 0.894 |
| *Bk. circumdata* | *H. binotatus* | 0.075 | 0.009 | 8.32 | **<.0001** | -0.094 | 0.022 | -4.31 | **0.000** |
| *Bk. circumdata* | *I. henselii* | 0.032 | 0.009 | 3.76 | **0.003** | -0.115 | 0.021 | -5.59 | **<.0001** |
| *Bk. circumdata* | *R. ornata* | 0.029 | 0.009 | 3.17 | **0.020** | -0.130 | 0.022 | -5.92 | **<.0001** |
| *Bk. hylax* | *H. binotatus* | 0.086 | 0.008 | 11.15 | **<.0001** | -0.120 | 0.019 | -6.39 | **<.0001** |
| *Bk. hylax* | *I. henselii* | 0.043 | 0.007 | 6.07 | **<.0001** | -0.141 | 0.017 | -8.20 | **<.0001** |
| *Bk. hylax* | *R. ornata* | 0.040 | 0.008 | 5.06 | **<.0001** | -0.156 | 0.019 | -8.13 | **<.0001** |
| *H. binotatus* | *I. henselii* | -0.043 | 0.005 | -7.84 | **<.0001** | -0.021 | 0.013 | -1.61 | 0.593 |
| *H. binotatus* | *R. ornata* | -0.046 | 0.006 | -7.44 | **<.0001** | -0.037 | 0.015 | -2.45 | 0.142 |
| *I. henselii* | *R. ornata* | -0.003 | 0.006 | -0.56 | 0.994 | -0.015 | 0.014 | -1.11 | 0.878 |

Supplementary Table 7. Taxonomy of unique sOTUs for each host species.

| Host | Phylum | Class | Order | Family | Genus | Species |
| --- | --- | --- | --- | --- | --- | --- |
| *Bo. faber* | Cyanobacteria | Synechococcophycideae | Pseudanabaenales | Pseudanabaenaceae | *Leptolyngbya* |  |
| *Bo. faber* | Proteobacteria | Betaproteobacteria | Burkholderiales | Comamonadaceae |  |  |
| *Bo. faber* | Proteobacteria | Betaproteobacteria | Burkholderiales | Comamonadaceae |  |  |
| *Bo. faber* | Firmicutes | Clostridia | Clostridiales | Lachnospiraceae |  |  |
| *Bo. faber* | Cyanobacteria |  |  |  |  |  |
| *Bo. faber* | Proteobacteria | Alphaproteobacteria | Rhizobiales | Methylocystaceae | *Methylosinus* |  |
| *Bo. faber* | Bacteroidetes | Bacteroidia | Bacteroidales | Bacteroidaceae | *Bacteroides* | *fragilis* |
| *Bo. faber* | TM6 | SJA-4 |  |  |  |  |
| *Bo. faber* | Actinobacteria | Actinobacteria | Actinomycetales |  |  |  |
| *Bo. faber* | Proteobacteria | Betaproteobacteria | Burkholderiales | Comamonadaceae | *Rubrivivax* | *gelatinosus* |
| *Bo. faber* | Actinobacteria | Actinobacteria | Actinomycetales | Mycobacteriaceae | *Mycobacterium* |  |
| *Bo. faber* | Proteobacteria | Betaproteobacteria | Burkholderiales | Comamonadaceae | *Limnohabitans* |  |
| *Bo. faber* | Proteobacteria | Betaproteobacteria | MWH-UniP1 |  |  |  |
| *Bo. faber* | Proteobacteria | Gammaproteobacteria | Enterobacteriales | Enterobacteriaceae |  |  |
| *Bo. faber* | Verrucomicrobia | Verrucomicrobiae | Verrucomicrobiales | Verrucomicrobiaceae | *Akkermansia* |  |
| *Bo. faber* | Verrucomicrobia | [Spartobacteria] | [Chthoniobacterales] | [Chthoniobacteraceae] | *Candidatus Xiphinematobacter* |  |
| *Bo. faber* | Proteobacteria | Betaproteobacteria | Neisseriales | Neisseriaceae | *Chromobacterium* |  |
| *Bo. faber* | Verrucomicrobia | Verrucomicrobiae | Verrucomicrobiales | Verrucomicrobiaceae | *Akkermansia* |  |
| *Bo. faber* | Firmicutes | Erysipelotrichi | Erysipelotrichales | Erysipelotrichaceae | *[Eubacterium]* | *dolichum* |
| *Bo. faber* | Proteobacteria | Deltaproteobacteria |  |  |  |  |
| *Bo. faber* | Fusobacteria | Fusobacteriia | Fusobacteriales | Fusobacteriaceae | *Cetobacterium* | *somerae* |
| *Bo. faber* | Acidobacteria | Solibacteres | Solibacterales | Solibacteraceae |  |  |
| *Bo. faber* | Proteobacteria | Betaproteobacteria | Burkholderiales | Comamonadaceae |  |  |
| *Bo. faber* | Proteobacteria | Betaproteobacteria | Rhodocyclales | Rhodocyclaceae | C39 |  |
| *Bo. faber* | Verrucomicrobia | Verrucomicrobiae | Verrucomicrobiales | Verrucomicrobiaceae |  |  |
| *Bo. faber* | Acidobacteria | Solibacteres | Solibacterales | Solibacteraceae |  |  |
| *Bo. faber* | Proteobacteria | Gammaproteobacteria | Enterobacteriales | Enterobacteriaceae |  |  |
| *Bo. faber* | Proteobacteria | Alphaproteobacteria | Rhizobiales |  |  |  |
| *Bo. faber* | Proteobacteria | Betaproteobacteria | Burkholderiales | Burkholderiaceae |  |  |
| *Bk. circumdata* | Proteobacteria | Gammaproteobacteria | Legionellales | Legionellaceae |  |  |
| *Bk. circumdata* | Proteobacteria | Gammaproteobacteria | Legionellales | Coxiellaceae | *Rickettsiella* |  |
| *Bk. hylax* | Chloroflexi | Ktedonobacteria | Thermogemmatisporales | Thermogemmatisporaceae |  |  |
| *Bk. hylax* | Bacteroidetes | [Saprospirae] | [Saprospirales] | Chitinophagaceae |  |  |
| *Bk. hylax* | Firmicutes | Clostridia | Clostridiales | Lachnospiraceae | *Coprococcus* |  |
| *H. binotatus* | Bacteroidetes | Flavobacteriia | Flavobacteriales | [Weeksellaceae] | *Wautersiella* |  |
| *H. binotatus* | Bacteroidetes | Bacteroidia | Bacteroidales | Bacteroidaceae | *Bacteroides* |  |
| *I. henselii* | Spirochaetes | Spirochaetes | Spirochaetales | Spirochaetaceae | *Treponema* |  |
| *I. henselii* | Bacteroidetes | Flavobacteriia | Flavobacteriales | Cryomorphaceae | *Fluviicola* |  |
| *I. henselii* | Bacteroidetes | Bacteroidia | Bacteroidales | Porphyromonadaceae | *Paludibacter* |  |
| *I. henselii* | Bacteroidetes | Bacteroidia | Bacteroidales | Porphyromonadaceae | *Paludibacter* |  |
| *I. henselii* | Proteobacteria | Deltaproteobacteria | Myxococcales |  |  |  |
| *I. henselii* | Proteobacteria | Gammaproteobacteria | Legionellales | Legionellaceae | *Legionella* |  |
| *I. henselii* | Bacteroidetes | Sphingobacteriia | Sphingobacteriales | Sphingobacteriaceae |  |  |
| *R. ornata* | Bacteroidetes | Bacteroidia | Bacteroidales |  |  |  |
| *R. ornata* | Firmicutes | Clostridia | Clostridiales | Lachnospiraceae |  |  |
| *R. ornata* | Firmicutes | Clostridia | Clostridiales | Lachnospiraceae | *Clostridium* |  |
| *R. ornata* | Proteobacteria | Betaproteobacteria | Burkholderiales | Comamonadaceae |  |  |
| *R. ornata* | Actinobacteria | Actinobacteria | Actinomycetales | Microbacteriaceae |  |  |
| *R. ornata* | Firmicutes | Erysipelotrichi | Erysipelotrichales | Erysipelotrichaceae |  |  |
| *R. ornata* | Firmicutes | Erysipelotrichi | Erysipelotrichales | Erysipelotrichaceae |  |  |
| *R. ornata* | Firmicutes | Clostridia | Clostridiales | Ruminococcaceae |  |  |
| *R. ornata* | Actinobacteria | Actinobacteria | Actinomycetales |  |  |  |
| *R. ornata* | Bacteroidetes | Sphingobacteriia | Sphingobacteriales | Sphingobacteriaceae |  |  |

Supplementary Table 8. Common core sOTUS for each host species, defined as the set of sOTUs detected in at least 90% of sampled individuals. Asterisk indicates core sOTUs that were also differentially abundant in the indicated host species. Some sOTUs could not be identified to all taxonomic ranks. No core sOTUs were detected for *Boana faber*.

| Phylum | Class | Order | Family | Genus | Species |
| --- | --- | --- | --- | --- | --- |
|  |  |  |  |  |  |
| ***Haddadus binotatus*** |  |  |  |  |  |
| * Proteobacteria | Alphaproteobacteria | Caulobacterales | Caulobacteraceae | *Mycoplana* |  |
| * Proteobacteria | Alphaproteobacteria | Rhizobiales | Bradyrhizobiaceae | *Bosea* |  |
| * Proteobacteria | Alphaproteobacteria | Rhizobiales | Hyphomicrobiaceae | *Devosia* |  |
| * Proteobacteria | Alphaproteobacteria | Rhizobiales | Hyphomicrobiaceae | *Devosia* |  |
| Proteobacteria | Alphaproteobacteria | Rhizobiales | Methylobacteriaceae | *Methylobacterium* |  |
| * Proteobacteria | Alphaproteobacteria | Rhizobiales | Rhizobiaceae | *Agrobacterium* |  |
| * Proteobacteria | Alphaproteobacteria | Rhizobiales | Rhizobiaceae | *Agrobacterium* |  |
| * Proteobacteria | Alphaproteobacteria | Rhizobiales | Rhizobiaceae | *Rhizobium* |  |
| * Proteobacteria | Alphaproteobacteria | Rhodobacterales | Rhodobacteraceae | *Paracoccus* | *marcusii* |
| *Proteobacteria | Alphaproteobacteria | Sphingomonadales | Sphingomonadaceae | *Novosphingobium* |  |
| Proteobacteria | Alphaproteobacteria | Sphingomonadales | Sphingomonadaceae | *Sphingomonas* | *asaccharolytica* |
| * Proteobacteria | Gammaproteobacteria | Xanthomonadales | Xanthomonadaceae |  |  |
|  |  |  |  |  |  |
| ***Ischnocnema henselii*** | |  |  |  |  |
| Proteobacteria | Gammaproteobacteria | Enterobacteriales | Enterobacteriaceae |  |  |
|  |  |  |  |  |  |
| ***Rhinella ornata*** |  |  |  |  |  |
| Bacteroidetes | Sphingobacteriia | Sphingobacteriales | Sphingobacteriaceae |  |  |
| Proteobacteria | Alphaproteobacteria | Rhizobiales | Rhizobiaceae | *Agrobacterium* |  |
| Proteobacteria | Gammaproteobacteria | Enterobacteriales | Enterobacteriaceae |  |  |
|  |  |  |  |  |  |
| ***Bokermannohyla circumdata*** | |  |  |  |  |
| * Proteobacteria | Betaproteobacteria | Burkholderiales | Alcaligenaceae |  |  |
|  |  |  |  |  |  |
| ***Bokermannohyla* hylax** | |  |  |  |  |
| * Proteobacteria | Betaproteobacteria | Burkholderiales | Alcaligenaceae |  |  |
| Proteobacteria | Gammaproteobacteria | Enterobacteriales | Enterobacteriaceae |  |  |
